# Effect of an integrated neonatal care kit on cause-specific neonatal mortality in rural Pakistan

**DOI:** 10.1080/16549716.2020.1802952

**Published:** 2020-08-25

**Authors:** Jessica Duby, Lisa G. Pell, Shabina Ariff, Amira Khan, Afsah Bhutta, Daniel S. Farrar, Diego G. Bassani, Masawar Hussain, Zulfiqar A. Bhutta, Sajid Soofi, Shaun K. Morris

**Affiliations:** a Department of Pediatrics, McGill University, Montreal, Canada; b Centre for Global Child Health, The Hospital for Sick Children, Toronto, Canada; c Center of Excellence in Women and Child Health, The Aga Khan University, Karachi, Pakistan; d Dalla Lana School of Public Health, University of Toronto, Toronto, Canada; e Department of Paediatrics, University of Toronto, Toronto, Canada

**Keywords:** Newborn, cause of death, verbal autopsy, community health workers, pakistan

## Abstract

**Background:**

In 2018, Pakistan had the world’s highest neonatal mortality rate. Within Pakistan, most neonatal deaths occur in rural areas where access to health facilities is limited, and robust vital registration systems are lacking. To improve newborn survival, there is a need to better understand the causes of neonatal death in high burden settings and engage caregivers in the promotion of newborn health.

**Objective:**

To describe the causes of neonatal death in a rural area in Pakistan and to estimate the effect of an integrated neonatal care kit (iNCK) on cause-specific neonatal mortality.

**Methods:**

We analyzed data from a community-based, cluster-randomized controlled trial of 5286 neonates in Rahim Yar Khan (RYK), Punjab, Pakistan between April 2014 and August 2015. In intervention clusters, Lady Health Workers (LHW) delivered the iNCK and education on its use to pregnant women while control clusters received the local standard of care. The iNCK included interventions to prevent and identify signs of infection, identify low birthweight (LBW), and identify and manage hypothermia. Verbal autopsies were attempted for all deaths. The primary outcome was cause-specific neonatal mortality.

**Results:**

Verbal autopsies were conducted for 84 (57%) of the 147 reported neonatal deaths. The leading causes of death were infection (44%), intrapartum-related complications (26%) and prematurity/LBW (20%). There were no significant differences in neonatal mortality due to prematurity/LBW (RR 0.43; 95% CI 0.15–1.24), infection (RR 1.10; 95% CI 0.58–2.10) or intrapartum-related complications (RR 1.04; 95% CI 0.0.45–2.41) among neonates who died in the intervention arm compared to those who died in the control arm.

**Conclusion:**

The major causes of neonatal deaths in RYK, Pakistan mirror the global landscape of neonatal deaths. The iNCK did not significantly reduce any cause-specific neonatal mortality.

## Background

In 2018, Pakistan had the highest neonatal mortality in the world with an estimated 42 neonatal deaths for every 1,000 live births [1]. Barriers to improving neonatal survival are most pronounced in rural regions where access to health care facilities is limited. In these communities, most primary health care is provided by community health workers, including those employed by the Lady Health Worker (LHW) Programme [2]. LHWs provide a range of home-based health promotion and disease prevention services in addition to maintaining birth records, managing minor illnesses, and identifying and referring sick community members. However, they do not attend childbirths and prompt visitation of newborns in the first few days of life and at other critical times has remained challenging [3,4]. Each LHW is responsible for approximately 1,000 people within a catchment area of about 100–150 houses, and many births continue to occur at home, often without skilled attendance [5]. Therefore, there is a need to empower mothers and families to provide important preventive care and to recognize signs of newborn illness early.

Estimates of the causes of neonatal mortality are required to identify health priorities and interventions. However, high burden countries where most neonatal deaths occur usually lack robust vital registration systems. For example, in Pakistan, most newborn deaths occur outside of the formal health care system and vital registration systems record less than 30% of all births and almost no deaths [6]. In such settings, formal autopsies are extremely rare, and medically certified causes of death are uncommon. As a result, verbal autopsies (VAs) – structured interviews with the deceased’s next of kin – are a feasible alternative and have become the international standard to determine the most likely cause of death for both research and surveillance purposes in such settings [7]. Determination of a cause of death is made following physician review, computer-based analysis, or a combination thereof, which are all commonly used methods of VA interpretation. When compared to medical record diagnoses, VAs have demonstrated a sensitivity ranging from 46 to 93% and a specificity ranging from 76 to 95% for neonatal causes of death [8–10].

**Figure 1.**
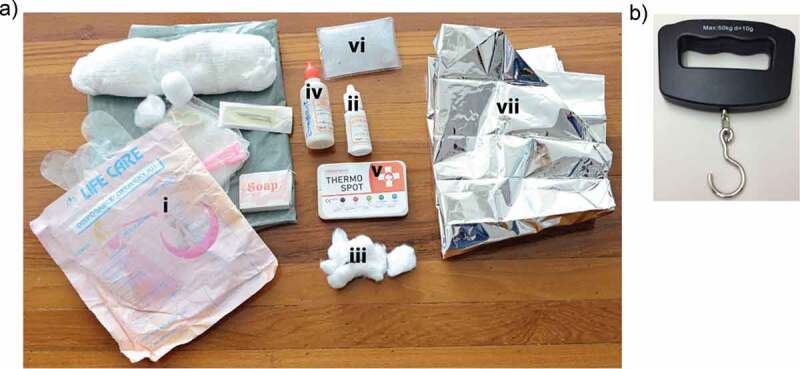
Integrated neonatal care kit.

A recent cluster-randomized controlled trial (cRCT) in Rahim Yar Khan (RYK), Punjab, Pakistan estimated the effect of delivering an integrated Neonatal Care Kit (iNCK) to pregnant women during a routine third trimester LHW visit on all-cause neonatal mortality [11,12]. LHWs taught women and families how to implement the iNCK, a simple package of evidence-based interventions, designed to improve neonatal health outcomes. The iNCK included a clean birth kit, 4% chlorhexidine, sunflower oil emollient, a temperature indicator sticker (Thermospot^TM^), a heat reflective polyester blanket and a reusable heat pack. In addition, LHWs in the intervention clusters were provided with a hand-held scale to weigh each newborn (Figure 1).

a) The integrated neonatal care kit (iNCK) included i) a clean birth kit; ii) 4% chlorhexidine solution that is to be applied with; iii) cotton balls; iv) sunflower oil emollient; v) Thermospot^TM^; vi) a reusable heat pack; and vii) a reflective polyester blanket. b) A hand-held scale was provided to the LHWs in the intervention clusters. Reproduced with permission from the iNCK study authors (12).

The provision of the iNCK reduced certain newborn morbidities related to infection and facilitated vital preventative care for low birthweight (LBW) infants [11]. Specifically, there was a 32% and 36% reduction in the risk of omphalitis and fever in the intervention arm compared to the control arm, respectively. In addition, the identification of LBW infants by LHWs among home-delivered infants was more than 3 times higher in the intervention arm compared to the control arm. Finally, the iNCK enabled caregivers to identify and respond to newborn hypothermia, a common complication among LBW and/or premature infants [11].

Despite the iNCK’s effect on the incidence and detection of clinical signs of neonatal infection and the identification of LBW infants, there was no significant reduction in all-cause neonatal mortality, the trial’s primary outcome, in the iNCK group compared to the control group [11]. However, it is plausible that the use of the iNCK reduced the risk of neonatal mortality only due to the morbidities addressed by the iNCK, namely infection and complications associated with being born LBW and/or prematurely. To investigate this possibility, we used VAs that were collected in the cRCT in RYK to estimate the effect of the iNCK compared to local standard of care on cause-specific neonatal mortality in RYK, Pakistan. Secondarily, we sought to provide an understanding of the underlying causes and contributing factors to neonatal deaths in RYK, Pakistan to inform future, targeted interventions.

## Methods

The study design, methods, and primary results of the iNCK cluster-randomized controlled trial in RYK, Pakistan have been previously reported [11,12]. In brief, a total of 150 clusters, defined as a village and its associated LHWs, were randomly assigned to receive either the iNCK (intervention) or local standard of care by LHWs (control). Clusters were stratified based on the number of LHWs serving each village. Between April 2014 to July 2015, 5451 pregnant women were enrolled (2663 and 2788 in the intervention and control clusters, respectively).

Data collectors visited households in both the intervention and controls clusters soon after birth (day 1) and up to four additional times during the first month of life (days 3, 7, 14, and 28). Data regarding demographics, pregnancy outcome, iNCK compliance and neonatal health and vital status were collected. If a stillbirth or neonatal death was identified, families were approached after a minimum grieving period of two weeks and verbal consent was obtained to administer a VA. Data collectors administering the VA had a minimum of 14 years of education and received training on how to conduct the structured interview in a simple and sensitive manner. The VA was administered to the mother if she was present or to the father if he was the only parent present. If neither parent was available, the data collector would attempt to visit at a later date or administer the VA to the most closely related adult caregiver who was present at the time of the interview.

The 2012 World Health Organization (WHO) VA Instrument was adapted and translated from English to Urdu [13]. The VA included categorical questions in addition to a free form narrative. The questions obtained information related to maternal health, pregnancy, delivery, the condition of the neonate after delivery, neonatal injuries, neonatal illnesses, treatments used and events leading up to the death (Supplemental Material). Responses to questions were collected on paper forms. Field supervisors validated 3% of VAs by re-administering and comparing responses between the original and re-administered versions. All forms were further reviewed by research medical officers for completeness and consistency prior to data entry.

The primary outcome was cause-specific neonatal mortality defined by the underlying cause of death. As per the International Classification of Diseases (ICD-10), the underlying cause of death is the disease or injury which initiated the sequence of events leading directly to death [14]. Additional antecedent conditions or morbidities that increased the risk for the underlying cause of death were documented as contributory causes of death. For example, if the reviewing physicians determined that an infant died due to an infection but the infant had also been born prematurely then infection would be considered as the underlying cause of death and prematurity as a contributory cause of death.

An underlying cause of death using was assigned by two physicians in Pakistan with a senior pediatrician adjudicating discrepancies. Each physician independently determined whether reported deaths were appropriately classified as neonatal deaths, as opposed to stillbirths or post-neonatal deaths, prior to assigning an underlying cause of death. A neonatal death was defined as a live born infant who died within 28 days of life (i.e., day 1–28). Subsequently, each VA was independently reviewed by a neonatologist and a pediatrician in Canada with a senior pediatrician adjudicating discrepancies. The additional review was undertaken to facilitate consideration for contributory causes of death. In order to identify contributory causes of death, the Canadian physicians, blinded to the Pakistani review team’s interpretations, independently re-assigned an underlying cause of death. For discrepant interpretations of the underlying cause of death between the Pakistani and the Canadian review teams, consensus was reached through discussion. All physician reviewers were educated in the principles of the WHO’s ICD-10 and all senior reviewers had prior experience with VA interpretation.

Causes of death were assigned using the coding manual from the Alliance for Maternal and Newborn Health Improvement (AMANHI) mortality study [15,16]. The AMANHI study used VAs to quantify the burden, timing and causes of maternal and neonatal deaths and stillbirths in sub-Saharan Africa and South Asia from 2012 to 2016. The AMANHI manual was created in accordance with the ICD-10 hierarchical classification system [14] and the WHO Verbal Autopsy Coding Standards [13].

For the current study, a list of programmatically relevant causes of neonatal death and their definitions were adapted from the AMANHI manual (Table 1). Given the difficulty of distinguishing between sepsis, pneumonia and meningitis in a neonate in a low resource setting, a single category of “*infection*’ was created to subsume all three diagnoses. In addition, the category labeled ‘*intrapartum-related complications*’ included both intrapartum asphyxia and birth injury because both causes of death are related to intrapartum management which would not be affected by the iNCK. Finally, given the low reliability of gestational age dating in the rural Pakistan study setting, prematurity and LBW were combined into one cause of death category labeled as ‘*prematurity/LBW*’.

**Table 1.** Potential underlying neonatal causes of death^a.^

Underlying Cause of Death	Abridged Case Definition
Infection	Generally well at birth and then develop poor feeding and/or temperature derangement and/or fast/difficulty breathing and/or lethargy and/or seizures. Includes sepsis, pneumonia and meningitis.
Intrapartum-related complications	Includes severe birth injury and perinatal asphyxia (failure to breathe at birth with progressively worsening condition or abnormal level of consciousness since birth).
Tetanus	Progressive inability to suck and stiffness starting after day 3 of life.
Congenital malformation	Major structural anomalies that directly impair normal respiratory, cardiac, feeding or gastrointestinal functions.
Prematurity/LBW	Death due to a complication specific to prematurity or gestational age < 34 weeks/birthweight < 2000 grams and no other underlying cause of death.
Accident/Injury	Severe accident/injury after birth directly resulting in death.
Other specific perinatal cause of death	Cause identified but not listed above.
Cause not able to be determined	Insufficient or poor quality VA data or physicians failed to reach consensus.

^a^Adapted from the Alliance for Maternal and Newborn Health Improvement (AMANHI) mortality study manual (15, 16)

Importantly, prematurity/LBW was primarily assigned as an underlying cause of death if a premature/LBW neonate died from a complication that was specific to prematurity, such as respiratory distress syndrome (RDS) or necrotizing enterocolitis (NEC). However, for any deaths without a clear cause of death, an underlying cause of death of prematurity/LBW was assigned if the gestational age at birth, based on self-reported last menstrual period, was less than 34 weeks or the birthweight was less than 2000 grams. Given the low reliability of gestational age dating in rural Pakistan, these gestational age and birthweight thresholds were selected to maximize the accuracy of a prematurity/LBW classification only if no other underlying cause was able to be identified [17]. In addition, for any deaths with another underlying cause of death, the same gestational age and birthweight thresholds were used to determine whether prematurity/LBW was a contributory cause of death.

The VA was the primary data source for cause of death determination. Where possible, VA responses were verified with data collected in other study questionnaires [11,12], and discrepancies were resolved using the best-available, supportive data. For example, precedence was given to the birthweight measured by data collectors over caregiver recall of the birthweight at the time of VA completion. In addition, when a caregiver-reported variable was collected more than once, the value reported most proximal in time to the event in question were used if the values were discrepant. For instance, gestational age was determined using the self-reported first day of last menstrual period which was recorded shortly after birth as opposed to the self-reported gestational age that was recalled at the time of VA completion. Hospital records or medical death certificates were not available for any of the reported deaths.

Maternal, delivery, and newborn characteristics were compared between deaths with a completed VA in the intervention arm and deaths with a completed VA in the control arm. Normally distributed and non-normally distributed continuous variables were assessed using clustered t-tests and Somers’ delta, respectively. Binary and categorical variables were analyzed using clustered chi-square tests.

Primary analyses of the effect of the intervention on underlying and contributory causes of death were conducted as complete case (i.e., participants for whom vital status was known at the end of the neonatal period), intention-to-treat irrespective of iNCK compliance. Per-protocol analyses were also performed in which a global compliance score, which took into account how closely each iNCK component was used according to its intended use, was ascribed to each participant. Global compliance scores were calculated using self-reported data on the utilization of each iNCK component as previously described [11]. In per-protocol analyses, cause-specific mortality rates were calculated at varying global compliance score threshold and were compared to a 1:1 propensity-score matched subset from the control arm. Propensity score matching was used to reduce bias due to potential confounding variables [18]. Propensity scores were generated within each treatment group and were based on maternal age, antenatal care and place of delivery. The denominator for the cause-specific mortality rates used in the per-protocol analysis were determined by the number of participants in the intervention arm who met the assigned compliance thresholds. For all analyses, there was no imputation of missing data. Risk ratios and 95% confidence intervals were calculated using log binomial regression and a robust variance estimator to account for clustering. Trend analysis was conducted using weighted linear regression. All statistical analyses were performed using Stata version 15 (StataCorp, College Station, TX).

## Results

**Figure 2.**
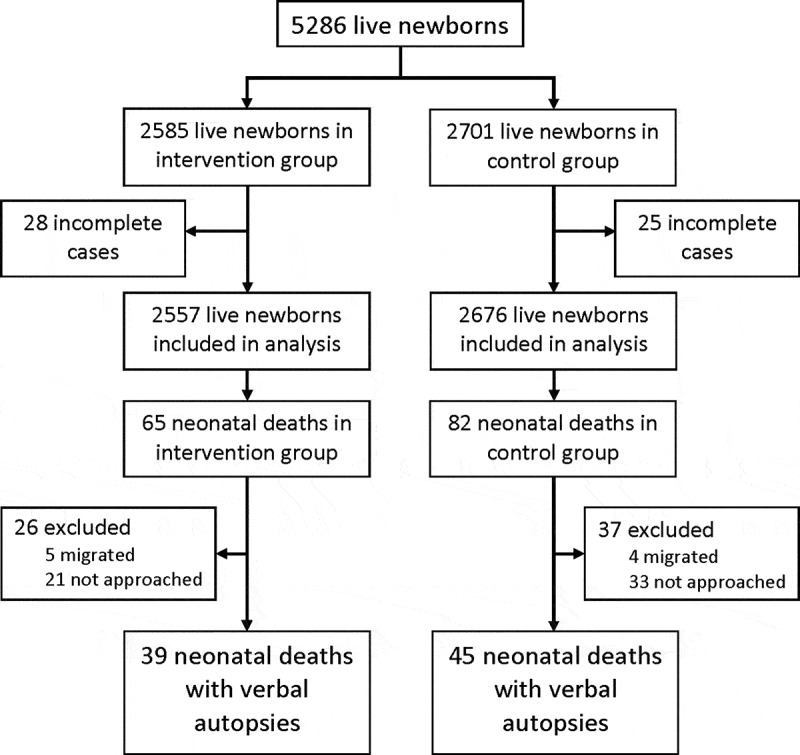
Trial profile.

Between April 2014 and August 2015, 5286 live births were delivered by women enrolled in the study (2585 in the intervention and 2701 in the control arm) (Figure 2) [11]. Neonatal outcomes were reported for 5233 newborns (99%), 2557 (98.9%) and 2676 (99.1%) in the intervention and control arms, respectively. In total, 147 neonatal deaths were reported, 65 in the intervention arm and 82 in the control arm [11].

Incomplete cases refers to all live newborns who were excluded from analysis due to any of the following reasions: withdrawn before day 28, lost to follow-up, migrated after delivery or field work concluded before day 28 (11).

VAs were completed for 84 (57%) neonatal deaths, 39 (60%) in the intervention arm and 45 (55%) in the control arm. Among the 63 (43%) neonatal deaths for which a VA was not completed, nine families migrated outside of the study catchment area following the neonatal death and were not reachable for further data collection. The remaining 54 families were not approached for a VA due to an implementation-related protocol deviation in which an abbreviated vital outcomes form, rather than a comprehensive VA, was administered to families with incomplete follow-up. There was no difference in place of residence at the level of the Union Council or place of birth between deaths with completed VAs and deaths without completed VAs. The median age at death for neonates with completed VAs was 4 days (IQR 3, 9) compared to 3 days (IQR 1, 3) for neonates without completed VAs (p < 0.001). Additional socio-demographic comparisons between deaths with completed VAs and deaths without completed VAs could not be performed due to missing data.

Among participants for which a neonatal VA was completed, 97% of mothers in the intervention clusters and 95% of mothers in the control clusters had at least one antenatal care visit (Table 2). Eighty-seven percent of mothers in the intervention clusters and 81% of mothers in the control clusters received at least one dose of tetanus toxoid during pregnancy. A greater proportion of the deceased neonates in the intervention clusters for whom a VA was completed were delivered at home compared to deceased neonates in the control clusters for whom a VA was completed (p = 0.04). Birthweights were measured by data collectors for 65% of all neonatal deaths with a completed VA, and the mean birthweight was 2299 grams in the intervention clusters and 2220 grams in the control clusters.

**Table 2.** Characteristics of participants for whom neonatal verbal autopsies were available, by treatment arm.

		Intervention	Control	*P* value
Maternal Characteristics				
N		38	43	–
Age (year), mean (SD)		28.4 (4.2)	30.0 (5.7)	0.41
Any antenatal care, n (%)		37 (97)	41 (95)	0.63
Tetanus toxoid during pregnancy, n (%)		33 (87)	35 (81)	0.51
Delivery Characteristics				
Place of delivery, n (%)				0.04
Home		16 (42)	9 (21)	–
Health Facility		22 (58)	34 (79)	–
Type of delivery, n (%)				0.70
Vaginal delivery		25 (66)	30 (70)	–
Caesarean-section		13 (34)	13 (30)	–
Delivery Attendant, n (%)^a^				0.11
Skilled		25 (66)	35 (81)	–
Unskilled		13 (34)	8 (19)	–
Newborn Characteristics				
N^b^		39	45	–
Male, n (%)		19 (49)	26 (58)	0.41
Gestational age at birth (weeks), median (IQR)		37 (35, 40)	36 (34, 38)	0.13
Birthweight (g), mean (SD)^c^		2299 (643)	2220 (585)	0.23
Medical treatment received prior to death, n (%)		25 (64)	32 (71)	0.49
Age at death, n (%)				0.51
Less 7 completed days		26 (67)	33 (73)	–
After 7 but before 28 completed days		13 (33)	12 (27)	–
Location of death, n (%)				0.78
Health Facility		17 (43)	21 (47)	–
In-transit to a health facility		3 (8)	5 (11)	–
Home		19 (49)	19 (42)	–

^a^Skilled delivery attendants included doctors, nurses, midwives and Lady Health Visitors. Unskilled delivery attendants included traditional birth attendants and family members.

^b^The intervention group included 36 singletons, 1 twin pair and 1 twin whose sibling survived the neonatal period. The control group included 40 singletons, 2 twin pairs and 1 triplet whose siblings survived the neonatal period.

^c^n = 27 and n = 28 in intervention and control group, respectively

**Figure 3.**
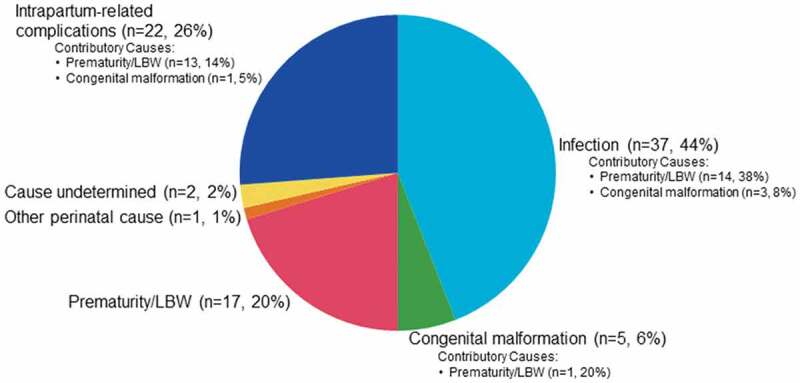
Cause of death for all verbal autopsies.

The most common underlying causes of death were infection (n = 37, 44%), intrapartum-related complications (n = 22, 26%) and prematurity/LBW (n = 17, 20%) (Figure 3). Five (6%) deaths were attributed to congenital malformation and one neonate (1%) died from another specific perinatal cause not listed in the coding manual. In two cases (2%), the underlying cause of death could not be ascribed. Twenty-two neonatal deaths had one associated contributory cause of death and one death had two associated contributory causes of death. Prematurity/LBW was the most common contributory cause of death (83%). Specifically, among deaths due to infection, prematurity/LBW was a contributory cause for 38% of these deaths. Prematurity/LBW contributed to 14% of deaths due to intrapartum-related complications, and among deaths due to congenital malformation, prematurity/LBW was a contributory cause for 14% of these deaths.

The risk of neonatal mortality due to infection as an underlying cause was not different between the intervention and control groups (RR 1.10; 95% CI 0.58–2.10) (Table 3). There was a 57% reduction in the risk of death due to prematurity/LBW in the intervention group compared to the control group; however, the result was not statistically significant (RR 0.43; 95% CI 0.15–1.24). There was also no difference in the risk of mortality attributed to intrapartum-related complications (RR 1.05; 95% CI 0.45–2.41) or congenital malformations (RR 1.57; 95% 0.26–9.39) between treatment groups.

**Table 3.** Underlying cause of death by treatment arm.

		Intervention	Control	Risk Ratio (95% CI)
Infection				
	Neonatal deaths, n	19	18	
	Neonatal mortality rate (per 1,000 live births)^a^	7.4	6.7	1.10 (0.58–2.1)
Intrapartum-related complications				
	Neonatal deaths, n	11	11	
	Neonatal mortality rate (per 1,000 live births)^a^	4.3	4.1	1.04 (0.45–2.41)
Prematurity/LBW				
	Neonatal deaths, n	5	12	
	Neonatal mortality rate (per 1,000 live births)^a^	2.0	4.5	0.44 (0.15–1.24)
Congenital malformation				
	Neonatal deaths, n	3	2	
	Neonatal mortality rate (per 1,000 live births)^a^	1.2	0.7	1.57 (0.26–9.39)

^a^Reported cause-specific neonatal mortality rates are minimum estimates given that a VA was not performed on 43% of deaths.

When stratified by age at death, the risk of dying from infection between days 1 and 7 of life (early neonatal death) was not statistically significant (RR 0.81; 95% CI 0.30–2.18). There was also no significant difference in the risk of dying from infection between days 8 and 28 of life (late neonatal deaths) between treatment groups (RR 1.39; 95% CI 0.59–3.30). All neonatal deaths due prematurity/LBW (n = 17) or congenital malformation (n = 5) and the majority of deaths due to intrapartum-related complications (n = 22, 86%) occurred in the early neonatal period.

In the per-protocol analysis, a trend was observed whereby the risk of mortality from infection, one of the main pathologies targeted by the iNCK, decreased in the intervention group compared to the control group as compliance increased (p = 0.01) (Table 4). When per-protocol analyses were conducted for causes of death that were not expected to be impacted by the iNCK (i.e. intrapartum-related complications and congenital malformations), a similar trend was not observed.

**Table 4.** Effect of the iNCK on cause-specific neonatal mortality by compliance cut-off score.

	Compliance cut-off score (%)	Participants per group^a^ (n)	Intervention Deaths (n)	Control Deaths (n)	Relative Risk (95% CI)
Infection					
	50	2160	16	17	0.94 (0.48–1.86)
	60	2073	15	17	0.88 (0.44–1.76)
	70	1985	15	17	0.88 (0.44–1.76)
	80	696	2	5	0.40 (0.08–2.06)
	90	647	1	5	0.20 (0.02–1.71)
	100	508	1	3	0.33 (0.03–3.20)
Prematurity/LBW					
	50	2160	4	12	0.33 (0.11–1.03)
	60	2073	2	12	0.17 (0.04–0.74)
	70	1985	0	11	–
	80	696	0	6	–
	90	647	0	5	–
	100	508	0	4	–
Congenital malformation or intrapartum-related complications^b^					
	50	2160	5	10	0.50 (0.17–1.46)
	60	2073	5	11	0.45 (0.16–1.30)
	70	1985	4	9	0.44 (0.14–1.44)
	80	696	2	3	0.67 (0.11–3.98)
	90	647	1	3	0.33 (0.03–3.20)
	100	508	1	2	0.50 (0.04–5.50)

^a^The number of participants that were included was determined by the number of participants in the intervention group who met the given cut-off compliance score. These participants were compared to a 1:1 propensity-score matched control group based on cluster assignment, maternal age, antenatal care and delivery location.

^b^Because none of the interventions in the iNCK targeted mortality from congenital malformations or intrapartum-related complications, these causes of death were combined for the compliance analyses.

## Discussion

In Pakistan, 251,000 neonates died in 2017 with the highest numbers in low-income populations in rural areas [1,19]. Understanding the major causes of neonatal death is an important first step towards identifying effective interventions to improve neonatal survival. This study demonstrated that the three main causes of neonatal death in a community-based, rural research setting in RYK, Pakistan are similar to the leading causes of neonatal deaths worldwide. Infection, intrapartum-related complications and prematurity/LBW accounted for 90% of all neonatal deaths in our study compared to 80% worldwide [7].

The interventions in the iNCK primarily targeted neonatal deaths due to infection through infection prevention measures and the early identification of danger signs, including fever and hypothermia. The use of clean birth kits has been shown to reduce the rate of all-cause neonatal mortality in low-income countries [20], and the application of chlorhexidine to the umbilical stump decreases the rate of omphalitis in low-income countries [21]. Sunflower emollient enhances the skin’s barrier function and has been shown to reduce the rate of hospital-acquired infections for premature infants [22]. In addition, as temperature instability is a critical indicator of possible serious bacterial infection [23], the inclusion of a temperature-monitoring sticker enables families to detect and seek medical treatment for a possible infection. Indeed, in the iNCK trial, the risk of fever and the risk of omphalitis were significantly reduced in the intervention group compared to the control group although the overall risk of severe infection, defined using a composite of signs and symptoms, was not different between groups [11].

Some of the interventions in the iNCK also facilitated improved community-based care of premature and/or LBW infants. As part of the iNCK, LHWs were equipped with a hand-held scale to identify and refer LBW newborns to health facilities in a timely manner. The iNCK also included interventions to identify and manage hypothermia which is a known risk factor for RDS, a potentially lethal complication of prematurity [24].

Despite the iNCK’s biologic plausibility to reduce neonatal deaths from infection and prematurity/LBW, the provision of the iNCK to pregnant women did not result in a statistically significant reduction of any underlying cause of neonatal mortality. The same factors which contributed to the absence of a significant reduction on all-cause mortality in the primary iNCK trial may have also dampened the iNCK’s effect on cause-specific mortality [11]. For example, the all-cause neonatal mortality rate in the control group was lower than anticipated, possibly due, at least in part, to frequent home visits by study personnel, irrespective of treatment group. In addition, the current study lacked statistical power to detect differences in cause-specific mortality rates because of a small sample size. VA data were not collected for 43% of all neonatal deaths due to an unplanned implementation-related protocol deviation. Specifically, data collectors did not administer a VA for the majority of participants who did not receive the regular schedule of postnatal data collection visits and for whom a neonatal death was identified near the end of the study. As a result, cause-specific mortality rates are likely an underestimate of the true cause-specific mortality rates. While there was no difference in VA completion rate by trial arm, infants with completed VAs died later compared to infants without completed VAs. Differences in age of death could affect the true distribution of causes of death given that deaths from prematurity/LBW and intrapartum-related complications tend to occur at an earlier age than deaths attributed to infection [17].

Because of limited statistical power in this study, further study of the iNCK’s effect on cause-specific mortality in a larger study and with more complete VA capture of deaths is required to understand if the iNCK reduces deaths from infection and/or prematurity/LBW. While there was not a statistically significant reduction in the risk of death from prematurity/LBW in the iNCK clusters compared to control clusters, the results may be clinically relevant. Simply identifying which newborns are premature/LBW is an essential first step in any efforts to reduce related mortality. As reported in the main iNCK trial, infants in the intervention arm who were born at home were almost five times as likely to be weighed by a LHW in the first three days of life compared to the control arm (p = 0.001) which may have facilitated early identification and referral of premature/LBW infants [11]. Despite improvements in the proportion of newborns weighed at home in the first three days of life in the intervention arm relative to the control arm, the overall proportion of home-delivered newborns in the intervention arm who were weighed within three days of life remained low (16.6%). Strategies to improve the likelihood that LHWs will weigh newborns in the first three days of life may increase the iNCK’s effect on survival of premature/LBW infants. Second, given that most of the infection-related interventions in the iNCK targeted early-onset, rather than late-onset, sepsis, a greater impact on deaths from infection in the first week of life rather than between days 8 and 28 was expected. While a significant difference in deaths attributed to infection during the first week of life in the intervention compared to control clusters was not observed, the direction of effect size was as expected (RR 0.81; 95% CI 0.30–2.18) and a larger study would provide the power to determine if this difference is likely to be real. Indeed, an understanding of the iNCK’s effect on cause-specific mortality in the early neonatal period is limited by the fact that VA data were disproportionately unavailable for early neonatal deaths compared to late neonatal deaths. Finally, a trend emerged whereby increasing compliance to the iNCK reduced the risk of neonatal deaths due to infection. Importantly, a similar trend was not observed for causes of death that lacked biologic plausibility to be reduced by the iNCK.

## Conclusion

Harnessing the power of parents, families and communities is the fourth strategic objective of the WHO’s action plan to end preventable neonatal death [25]. In this study, empowering families and community health workers through providing packaged interventions to prevent and identify neonatal infection as well as identify LBW infants did not significantly reduce cause-specific mortality. However, this may be in part due to challenges faced by caregivers and LHWs in using the iNCK as intended as well as a small sample size. Further research should focus on improving compliance to the iNCK.

## Supplementary Material

Supplemental Material

## Paper context

A recent trial in rural Pakistan showed that a neonatal intervention package implemented by families and community health workers reduced the risk of omphalitis and fever and increased the identification of low birthweight infants. However, the package of interventions did not significantly reduce the risk of neonatal death from any specific cause. Further research to empower families and communities in reducing neonatal mortality from infection and prematurity is needed.
