# Supplementary material for: Effect of an integrated neonatal care kit on cause-specific neonatal mortality in rural Pakistan
Source: Glob Health Action. 2020 Aug 25;13(1):1802952. doi: 10.1080/16549716.2020.1802952 (PMC7480452; doi:10.1080/16549716.2020.1802952)
Supplement: Supplemental Material [file ZGHA_A_1802952_SM4216.docx]

**Neonatal Verbal Autopsy Form**

نوزائیدہ کا وربل آٹوپسی فارم

| **SN** | **Section A- Basic and Identification Information** سیکشن اےبنیادی اور شناختی معلومات | |
| --- | --- | --- |
| 1 | Child and Mother ID (Household ID)  ماں اور بچے کا شناختی نمبر(گھر کا شناختی نمبر) | ماں اور بچے کا شناختی نمبرلکھیں |
| 2 | Village name and IDگاٗوٗں کا نام اور شناختی نمبر | گاٗوٗں کا نام اور شناختی نمبر لکھیں |
| 3 | UC name and IDیونین کونسل کا نام اور شناختی نمبر | یونین کونسل کا نام اور شناختی نمبر لکھیں |
| 4 | Date of visit دورے کی تاریخ | DD MM YYYY  دن مہینہ سال |
| 5 | Date of outcome نتیجے کی تاریخ | DD MM YYYY  دن مہینہ سال |
| 6 | Was the verbal consent obtained?  کیا زبانی رضامندی حا صل کی گٗیٗ؟ | 1=Yes, 2=No-Stop and do not continue  1=ہاں, 2=نہیں |
| 7 | Was the mother available for interview?  کیا ماں انٹرویو کے لیے موجود تھی؟ | 1=Yes, 2=No (if yes then proceed to question 9 and if no then ask question 8)  1=ہاں, 2=نہیں  (اگر ہاں تو سوال نمبر۹ پر جائیں اگر نہیں تو سوال نمبر ۸ پو چھیں) |
| 8 | Relation of the respondent to the child  جواب دہندہ کا بچے سے تعلق | 1=grand motherدادی  2=auntخالہ،پھوپھی،تائی،چچی  3=sister بہن  4=nanny نانی  5=another relationکوئی اوررشتہ |

| 9 | Visit Outcome:  دورے کا نتیجہ | | | 1=Completed مکمل  2=Not at Homeگھر پر نہیں تھا  3=Postponed تاخیر  4=Refused انکار کر دیا  5=Partly Completed  ادھورا مکمل ہوا  6=No appropriate respondent found  مناسب جواب دہندہ نہ ملا  7=Other (Specify)  دیگر (واضح کریں) | |
| --- | --- | --- | --- | --- | --- |
| Verbal Consent  Hello, My name is ______________________________________________ and I am working with Neonatal Birth Kit project in which the baby was enrolled. We are very sorry that the baby has died and the loss that you and the family are experiencing. Part of this research study is determining why babies die in this area so we are collecting information on the causes of death of the newborn. This information is important to identifying the major causes of newborn deaths in this area and will be used to improve the health of this population. The questions in this survey will help our researchers determine why your baby died. Some of the questions we ask may be upsetting and you do not have to answer any questions that you do not wish to. We would very much appreciate your participation in this effort. We want to ask you about the circumstances leading to the death of the deceased. Participation in this survey is voluntary. Whatever information you provide will be kept strictly confidential. No information identifying you or the deceased will ever be released to anyone outside of this information collection activity. Participation in this survey is voluntary and you can choose not to answer any individual question or all of the questions. You may also choose to stop the interview at any time without any consequences at all. However we hope that you will participate in this survey since the results will help the government improve services for people and children. The survey will take about 20 to 25 minutes to complete.  At this time, do you want to ask me anything about the purpose or content of this interview?  May I begin the interview now?  Signature of the interviewer ___________________________Date \|___\|___\|/\|___\|___\|/\|___\|___\|  اسلام و علیکم،  میرا نام ہے اور میں نیونیٹل برتھ کٹ پراجیکٹ میں کام کر رہی ہوں۔ہم اس بچے،بچی کی معلومات اکھٹی کر رہے ہیں جو اس مطالعے میں شامل تھا اور انتقال کر گیا۔ہم آپ کی اس میں شمولیت کی کوشش کو بہت سراہیں گے۔ ہم آپ سے وہ حالات جاننا چاہیں گے جن کی وجہ سے بچے کا انتقال ہوا۔ آپ ہمیں جو بھی معلومات فراہم کریں گی ہم اسے صیغہ راز میں رکھٰیں گے۔آپ کے بارے میں یا مرحوم بچے کے بارے میں کوئی بھی معلومات اس مطالعے سے باہر کسی کو نہیں دی جائے گی۔ اس سروے میں آپ کی شمولیت رضا کا رانہ ہے اور آپ کی مرضی ہے کہ ان میں سے کچھ یا تمام سوالات کے جواب نہ دیں۔آپ بغیر کسی جھجھک کے یہ انٹرویو کسی بھی وقت روک سکتی ہیں۔ تاہم ہم آپ سے یہ امید کرتے ہیں کہ آپ اس سروے میں شمولیت کریں گیجس کی وجہ سے گورنمنٹ کو لوگوں اور بچوں کیلئے سہولیات کی بہتر فراہمی میں مدد ملے گی۔  اس وقت،اس انٹرویو کے بارے میں کوئی سوال پوچھنا چاہیں گی؟  کیا میں اب انٹرویو شروع کر سکتی ہوں؟  انٹرویو لینے والے کے دستخط: تاریخ: | | | | | |
| 10 | | Respondent agrees to be interviewed  کیا جواب دہندہ ایٹرویو دینے کیلئے راضی ہے؟ | 1=Yes ہاں  2=No-Stop and do not continue نہیں | | 🡪STOP |

| SN | Questions | Responses | | | Skip | |
| --- | --- | --- | --- | --- | --- | --- |
| **2. Basic information about respondent** جواب دہندہ کے بارے میں بنیادی معلومات | | | | | | |
| 11 | Record the time at start of interview  انٹرویو شروع کرنے کے وقت کا اندراج کریں | Hours \|___\|___\|  گھنٹے  Minutes \|___\|___\|  منٹس | | |  | |
| 12 | What is your relationship to deceased?  مرحوم سے آپ کا کیا تعلق تھا؟ | 1=Mother [Preferred]ماں  2=Fatherباپ  3=Grandmotherدادی  4=Aunt خالہ،تائی،پھوپھی، چچی  5=Sister بہن  6=Grandfather دادا  7=Uncle خالو،تایا،چچا،پھپھا،ماموں  8=Brother بھائی  9=Other (Specify دیگر(واضح کریں) | | | 🡪14  🡪14 | |
| 13 | Respondent initials and year of birth | Initials……………………...\|___\|___\|__ \|  Birth Year……………..\|___\|___\|___\|___\| | | |  | |
| 14 | Did you live with the deceased in the period leading to her/his death?  کیا آپ مرحوم کے ساتھ اس عرصے میں اس کے ساتھ رہیں جس میں اس کی وفات ہوئی؟ | 1=Yes ہاں  2=No نہیں | | |  | |
| **3. Information on the deceased and date/place of death**  مرحوم کی وفات کی جگہ اور تاریخ کے بارے میں معلومات | | | | | | |
| 15 | What was the sex of deceased?  مرحوم کی کیا جنس تھی؟ | | 1=Female لڑکی  2=Male لڑکا  3=Any otherکوئی اور | |  | |
| 16 | When was the deceased born?  مرحوم کب پیدا ہوا؟ | | Days دن \|___\|___\|  Months مہینے \|___\|___\|  Year سال \|___\|___\|___\|___\| | |  | |
| 17 | How old was the deceased when she/he died?  موت کے وقت مرحوم کی کیا عمر تھی؟ | | Age in Days دنوں میں عمر \|___\|___\| | |  | |
| 18 | When did she/he die?  کب فوت ہوا،ہوئی؟ | | Days دن \|___\|___\|  Months مہینے \|___\|___\|  Year سال \|___\|___\|___\|___\| | |  | |
| 19 | Where did s/he die?  کہاں فوت ہوا،ہوئی؟ | | 1= Home گھر  2= Public Hospital سرکاری ہسپتال  3= Private Hospital پرائیویٹ ہسپتال  4= Maternity Centre مٹرنٹی سنٹر  5= Clinic کلینک  6= Other  Specify دیگر ( واضح کریں) ____________________________ | |  | |
| 20 | How old was the baby when the fatal illness started? ***00=less than 24hrs after birth***  بچے کی عمر کیا تھی جب اسے مہلک بیماری شروع ہوئی؟  00 لکھیں اگر پیدائیش کے بعد 24 گھنٹوں سے کم ہوتو۔ | | 1=Days..............................دن..\|___\|___\|  2=Don’t Knowمعلوم نہیں  3=Not applicable قابل اطلاق نہیں | |  | |
| 21 | For how long was s/he ill before s/he died OR How long did the fatal illness last (in days)? ***00=less than 24hrs after birth***  ***وفات سے پہلے بچہ کتنا عرصہ بیمار رہایا اسے مہلک بیماری کتنا عرصہ رہی. (دنوں میں)***  00 لکھیں اگر پیدائیش کے بعد 24 گھنٹوں سے کم ہوتو | | 1=Days..............................دن..\|___\|___\|  2=Don’t Knowمعلوم نہیں  3=Not applicable قابل اطلاق نہیں | |  | |
| **4. Respondent’s account of illness/events leading to death** جواب دہندہ کے مطابق موت کی وجوہات | | | | | | |
| 22 | Could you tell me about the illness/events that led to her/his death?  کیا آپ مجھے مرحوم کی بیماری یا ان مواقعوں کے بارے میں بتا سکتی ہیں جن کی وجہ سے موت واقع ہوئی؟  _______________________________________________________________________________  _______________________________________________________________________________  _______________________________________________________________________________­­­­­  _______________________________________________________________________________  _______________________________________________________________________________  _______________________________________________________________________________  _______________________________________________________________________________  _______________________________________________________________________________  _______________________________________________________________________________ | | | | | |
| 23 | Cause of death 1 according to respondent  جواب دہندہ کے مطابق موت کی پہلی وجہ  _______________________________________________________________________________ | | | | | |
| 24 | Cause of death 2 according to respondent  جواب دہندہ کے مطابق موت کی دوسری وجہ  _______________________________________________________________________________ | | | | | |
| 5. Pregnancy history حمل کی معلومات | | | | | | |
| I would like to ask you some questions concerning the mother and symptoms that the deceased had/showed at birth and shortly after. Some of these questions may not appear to be directly related to the baby’s death. Please bear with me and answer all the questions. They will help us to get a clear picture of all possible symptoms that the deceased had.  میں آپ سے ماں اور مرحوم کی ان علامتوں کے بارے میں جو اسے پیدائش اور اس کے فورا بعد ہوئیں کے بارے میں کچھ سوالات کرنا چاہوں گی۔ان میں سے کچھ سوالات شاید براہ راست بچے کی موت سے تعلق نہ رکھیں۔ برائے مہربانی مجھے برداشت کریں اور سب سوالات کے جواب دیں۔ان سے ہمیں ان تمام علامات کے بارے میں جانکاری ہو گی جن کی وجہ سے مرحوم کی موت ہوئی۔ | | | | | | |
| 25 | How many births including stillbirths, did the mother have before this baby?  اس بچے سے پہلے ماں کتنے بچوں کو جنم دے چکی ہے بشمول مردہ پیدائش؟ | 1=Number of Births(Still births)\|___\|___\|  پیدائشوں کی تعداد،مردہ پیدائشیں  2=Don’t know پتہ نہیں | | | |  |
| 26 | How many months was the pregnancy when the baby was born?  حمل کی کتنی مدت پر بچے کی پیدائش ہوئی؟ | Months مہینے \|___\|___\|  Don’t know پتہ نہیں | | | |  |
| 27 | Did the pregnancy end earlier than expected?  کیا حمل مقررہ مدت سے پہلے ختم ہوگیا؟ | 1=Yes ہاں  2=No نہیں  3=Don’t know پتہ نہیں | | | | 🡪29  🡪29 |
| 28 | How many weeks before expected date of delivery?  حمل کی مکمل مدت سے کتنے ہفتے پہلے؟ | 1=Weeks ہفتے \|___\|___\|  2=Don’t know پتہ نہیں | | | |  |
| 29 | During pregnancy did the mother suffer from any of the known illnesses?  کیا اس حمل میں ماں ان میں سے کسی بیماری کا شکار ہوئی؟ | 1= High Blood Pressure بلند فشار خون  2= Heart Disease دل کی بیماری  3= Diabetes ذیابطیس  4= Epilepsy/Convulsion مرگی،جھٹکے  5= Malaria (recently) ملیریا (حالیہ دنوں میں)  6= Depression اعصابی دباو  7= Stroke صدمہ  8 = Don't know پتہ نہیں  9= Other(Specify)  دیگر ( واضح کریں)  _____________________  10= No complication  کوئی بیماری نہیں تھی | | | |  |
| 30 | Did she have a recent test for malaria?  کیا حالیہ دنوں میں اس نے ملیریا کا ٹیسٹ کروایا؟ | 1=Yes ہاں  2=No نہیں  3=Don’t know پتہ نہیں | | | | 🡪32  🡪32 |
| 31 | What was the result of the malaria test?  ملیریا ٹیسٹ کا کیا نتیجہ نکلا؟ | 1= Positive مثبت  2= Negative منفی  3= Don’t know معلوم نہیں | | | |  |
| 32 | During the last 3 months of pregnancy did the mother suffer from any of the following illnesses?  کیا حمل کی آخری سہ ماہی میں ماں ان میں سے کسی بیماری کا شکار ہوئی؟ | 1= Vaginal Bleeding فرج سے خون کا اخراج  2= Smelly Vaginal Discharge  فرج سے بدبو دار مواد کا اخراج  3= Puffy face منہ پہ سوجن  4= Headache سردرد  5= Blurred Vision دھندلا نظر آنا  6= Convulsion دورے / جھٹکے  7= Febrile Illness بخار زدہ  8= Severe abdominal pain (Not labor pain)  شدید پیٹ درد (درد زہ نہیں)  9= Severe Anemia خون کی شدید کمی  10= Pallor or shortness of breath (Both)  پیلا پن / سانس لینے میں دشواری (دونوں)  11=Edema or Swelling of Lower Limbs  ٹانگوں پہ سوجن یا ورم  12_=_ Don't know معلوم نہیں  13= Other (Specify) دیگر (وضاحت کریں)  __________________________  14= No complication کوئی پیچیدگی نہیں ہوئی | | | |  |
| 33 | Was the baby alive at the time of delivery?  کیا بچہ پیدائیش کے وقت زندہ تھا؟ | 1=Yes ہاں  2=No نہیں  3=Don’t know پتہ نہیں | | | |  |
| 34 | Was the child a single or multiple births?  کیا بچے کی پیدائش واحد تھی یا جڑواں پیدائش تھی؟ | 1=Singleton واحد  2=Twin جڑواں  3=Triplet or moreتین یا اس سے ذیادہ  4=Don’t Know پتہ نہیں | | | | 🡪36  🡪36 |
| 35 | If multiple births then what was the birth order of the child that died?  جس بچے کا انتقال ہوا پیدائش میں اس کا کون سا درجہ تھا؟ | 1=First پہلا  2=Second دوسرا  3=Third or higher تیسرا یا اس سے زیادہ  4=Don’t know پتہ نہیں | | | |  |
| **6. Delivery History** زچگی کے بارے میں معلومات | | | | | | |
| 36 | Where was the child born (NAME)?  بچہ کہاں پیدا ہوا( نام)؟ | 1=Home **گھر**  2=Dispensary **ڈسپنسری**  3=Maternity home **میٹرنٹی ہوم**  3=Clinic **کلینک** 4=Basic health unit**بنیادی مرکز صحت** 5=RHC  **دیہی مرکز صحت** 6=THQ **تحصیل ہیڈ کوارٹر**  7=DHQ **ڈسٹرکٹ ہیڈ کوارٹر**  8=Private clinic **پرایئویٹ کلینک**  9=Private hospital **پرائیویٹ ہسپتال**  10= on the way **راستے میں**  11=Other (specify) **دیگر ( واضح کریں)** ____________________________ | | | |  |
| 37 | Who conducted the delivery?  زچگی کس نے کروائی؟ | 1=No one **کسی نے نہیں**  2=Family member **گھر کے فرد نے**  3=Traditional Birth Attendant **دائی**  4=Lady Health Worker **لیڈی ہیلتھ ورکر**  5=Lady Health Visitor **لیڈی ہیلتھ وزیٹر**  6=Midwife **مڈ وائف**  7= Nurse **نرس**  8= Government Doctor **گورنمنٹ ڈاکٹر**  9=Private Doctor **پرائویٹ ڈاکٹر**  10= Neighbor **ہمسائی**  11=Don’t know **معلوم نہیں** 12=Other (specify) **دیگر ( واضح کریں)** | | | |  |
| 38 | When did the water break?  پانی کب آنا شروع ہوا؟ | 1=Before labor started  زچگی شروع ہونے سے پہلے  2=During labor زچگی کے دوران  3=Don’t know پتہ نہیں | | | |  |
| 39 | How many hours after the water broke was the baby born?  پانی آنے کے کتنی دیر بعد بچہ پیدا ہوا؟ | 1=Less than 24 Hours۲۴ گھنٹے سے پہلے  2=24 Hours or more۲۴ گھنٹے کے بعد  3=Don’t know پتہ نہیں | | | |  |
| 40 | What was the colour of the liquid when the water broke?  جب پانی آنا شروع ہوا تو اسکی رنگت کیسی تھی؟ | 1= Clear/Normal صاف / نارمل  2= Green/brown سبز / بھورا  3= Don’t know معلوم نہیں | | | |  |
| 41 | Did the water smell foul?  کیا پانی میـں سے بدبو آرہی تھی؟ | 1=Yes ہاں  2=No نہیں  3=Don’t know پتہ نہیں | | | |  |
| 42 | Did the baby stop moving in the womb?  کیا بچے نے بچہ دانی میں حرکت کرنا روک دی تھی؟ | 1=Yes ہاں  2=No نہیں  3=Don’t know پتہ نہیں | | | | 🡪45  🡪45 |
| 43 | When did the baby stop moving in the womb?  بچے نے بچہ دانی میں حرکت کرنا کب روکی؟ | 1=Before labor started  زچگی شروع ہونے سے پہلے  2=During labor زچگی کے دوران  3=Don’t know پتہ نہیں | | | | 🡪45  🡪45 |
| 44 | How long before labor did the baby stop moving? (Hours OR days)  زچگی سے کتنا عرصہ پہلے بچے نے حرکت کرنا روک دی تھی؟ (گھنٹے یا دن) | 1=Hours **گھنٹے**  \|___\|___\|  2=Days دن \|___\|___\|  3= Don’t knowمعلوم نہیں | | | |  |
| 45 | Did the birth attendant listen for fetal heart sounds during labor?  کیا زچگی کروانے والی نے زچگی کے دوران بچے کے دل کی دھڑکن سنی؟ | 1=Yes ہاں  2=Noنہیں  3=Don’t know پتہ نہیں | | | | 🡪47  🡪47 |
| 46 | Were fetal heart sounds present?  کیا بچےکی دھڑکن موجود تھی؟ | 1=Yes ہاں  2=Noنہیں  3=Don’t know پتہ نہیں | | | |  |
| 47 | Was there excess bleeding on the day labor started?  کیا زچگی کے دن زیادہ خون بہنا شروع ہو گیا تھا؟ | 1=Yes ہاں  2=Noنہیں  3=Don’t know پتہ نہیں | | | |  |
| 48 | Was there excessive bleeding before labor started?  کیا زچگی سے پہلے خون کا زیادہ اخراج ہوا تھا۔ | 1=Yes ہاں  2=No نہیں  3=Don’t know معلوم نہیں | | | |  |
| 49 | Was there excessive bleeding during labor?  کیا زچگی کے دوران خون کا زیادہ اخراج ہواتھا؟ | 1=Yes ہاں  2=No نہیں  3=Don’t know معلوم نہیں | | | |  |
| 50 | Was there excessive bleeding after delivering the baby?  کیا بچے کی پیدائیش کے بعد خون کا زیادہ اخراج ہوا تھا؟ | 1=Yes ہاں  2=No نہیں  3=Don’t know معلوم نہیں | | | |  |
| 51 | Did the mother have fever on the day labor started?  کیا ماں کو زچگی کے دن بخار تھا؟ | 1=Yes ہاں  2=Noنہیں  3=Don’t know پتہ نہیں | | | |  |
| 52 | Did the mother have fever during the labor?  کیا زچگی کے دوران ماں کو بخار ہوا؟ | 1=Yes ہاں  2=No نہیں  3=Don’t know معلوم نہیں | | | |  |
| 53 | How long did the labor pain last?  درد زہ کتنی دیر رہا؟ | 1=Less than 12 Hours۱۲ گھنٹوں سے کم  2=12-23 Hours۱۲۔۲۳ گھنٹے  3=24 Hours or more۲۴ گھنٹوں سے زیادہ  4=Don’t know پتہ نہیں | | | |  |
| 54 | Was the mother in labor for unusually long (more than 24 hours)?  کیا ماں خلاف معمول زیادہ عرصہ کیلیئے درد زہ میں رہی (24 گھنٹوں سے زیادہ)؟ | 1=Yes ہاں  2=No نہیں  3=Don’t know معلوم نہیں | | | |  |
| 55 | Did the mother receive any injection during the labor but before the delivery?  کیا زچگی کے دوران مگر پیدائیش سے پہلےماں کو کوئی ٹیکہ لگا؟ | 1=Yes ہاں  2=No نہیں  3=Don’t know معلوم نہیں | | | |  |
| 56 | Was it a normal vaginal delivery?  کیا یہ نارمل زچگی تھی؟ | 1=Yes ہاں  2=Noنہیں  3=Don’t know پتہ نہیں | | | | 🡪58  🡪58 |
| 57 | What type of delivery was it?  یہ کس قسم کی زچگی تھی؟ | 1=Vaginal delivery with Episiotomy  چھوٹا آپریشن  2=Forceps/Vacuum  اوزار کے زریعے کی گئی زچگی  3=Caesarean section بڑا آپریشن  4=Other (Specify)__________________  دیگر ( واضح کریں)  5=Don’t Know پتہ نہیں | | | | **🡪**60 |
| 58 | Which part of the baby came first?  بچے کا کون سا حصہ پہلے آیا؟ | 1=Head سر  2=Bottom دھڑ  3=Feetپاؤں  4=Arm/Hand بازو، ہاتھ  5=Other (Specify)__________________  دیگر ( واضح کریں)  6=Don’t Know پتہ نہیں | | | |  |
| 59 | Did the umbilical cord come out before the baby was born?  کیا بچے کی ناڑو اس کی پیدائش سے پہلے باہر آگئی تھی؟ | 1=Yes ہاں  2=Noنہیں  3=Don’t know پتہ نہیں | | | |  |
| **7. Condition of the baby soon after birth** | | | | | | |
| 60 | At birth what was the size of the baby?  پیدائش کے وقت بچے کی کیا جسامت تھی؟ | 1=Smaller than normal نارمل سے کم  2=Normal نارمل  3=Larger than normal نارمل سے زیادہ  4=Don’t know پتہ نہیں | | |  | |
| 61 | Was the baby premature?  کیا بچہ وقت سے پہلے پیدا ہو گیا تھا؟ | 1=Yes ہاں  2=Noنہیں  3=Don’t know پتہ نہیں | | | 🡪63  🡪63 | |
| 62 | How many months or weeks along was the pregnancy?  حمل کو کتنے ماہ اور ہفتے ہو گئے تھے؟ | 1=Months مہینے \|___\|___\|  2=Weeks ہفتے \|___\|___\|  3=Don’t know پتہ نہیں | | |  | |
| 63 | What was the birth weight of the baby?  پیدائش کے وقت بچے کا کیا وزن تھا؟ | 1=Grams کلوگرام \|___\|___\|.\|___\|___\|  2=Don’t know پتہ نہیں | | |  | |
| 64 | How was the cord cut?  ناڑو کیسے کاٹا گیا؟ | 1=Sterile blade **جراثیم سے پاک بلیڈ سے**  2=Other(specific) **دیگر ( واضح کریں)**  3=Don’t know **معلوم نہیں** | | |  | |
| 65 | Was anything applied to the umbilical cord stump after birth?  کیا پیدائش کے بعد ناڑو پر کچھ لگایا گیا؟ | 1=Yes ہاں  2=Noنہیں  3=Don’t know پتہ نہیں | | | 🡪67  🡪67 | |
| 66 | What was it?  کیا لگایا؟ | _________________________________________  (Specify) ( واضح کریں) | | |  | |
| 67 | Where there any signs of injury or broken bones?  کیا کسی زخم یا ٹوٹی ہوئی ہڈی کا نشان تھا؟ | 1=Yes ہاں  2=Noنہیں  3=Don’t know پتہ نہیں | | | 🡪69  🡪69 | |
| 68 | Where were the marks or signs of injury?  زخم کے نشان کہاں تھے؟ | _________________________________  _________________________________  (Specify) ( واضح کریں) | | |  | |
| 69 | Was there any sign of paralysis?  کیا معزوری کی کوئی علامت تھی؟ | 1=Yes ہاں  2=Noنہیں  3=Don’t know پتہ نہیں | | |  | |
| 70 | Did the baby have any malformation?  کیا بچے میٰں کسی قسم کا نقص تھا؟ | 1=Yes ہاں  2=Noنہیں  3=Don’t know پتہ نہیں | | | 🡪72  🡪72 | |
| 71 | What kind of malformation did the baby have?  بچے میں کس قسم کا نقص تھا؟ | 1=Swelling/Defect on the back  سوجن یا کمر میں نقص  2=Very large head بہت بڑا سر  3=Very small head بہت چھوٹا سر  4=Defect of lip and/or Palate  ہونٹ یا تالو کا نقص  5=Other (Specify)___________  دیگر ( واضح کریں)  6=Don’t Know پتہ نہیں | | |  | |
| 72 | What was the color of the baby at birth?  پیدائش کے وقت بچے کا کیا رنگ تھا؟ | 1=Normal نارمل  2=Pale زرد  3=Blue نیلا  4=Don’t Know پتہ نہیں | | |  | |
| 73 | Was the cord wrapped several times around the neck of the child?  کیا ناڑو بچے کی گردن کے گرد بل دار انداز میں لپٹا ہوا تھا؟ | 1=Yes ہاں  2=No نہیں  3=Don’t know معلوم نہیں | | |  | |
| 74 | Did the baby breathe after birth, even a little?  کیا پیدائش کے بعد بچے نے کوئی سانس لیا؟ | 1=Yes ہاں  2=Noنہیں  3=Don’t know پتہ نہیں | | |  | |
| 75 | Was the baby given assistance to breathe?  کیا بچے کی سانس لینے میں مدد کی گئی؟ | 1=Yes ہاں  2=Noنہیں  3=Don’t know پتہ نہیں | | |  | |
| 76 | Did the baby ever cry after birth, even a little?  کیا بچہ پیدائش کے بعد تھوڑا سا بھی رویا؟ | 1=Yes ہاں  2=Noنہیں  3=Don’t know پتہ نہیں | | |  | |
| 77 | Did the baby move after birth, even a little?  کیا بچے نے پیدائش کے بعد کوئی حرکت کی؟ | 1=Yes ہاں  2=Noنہیں  3=Don’t know پتہ نہیں | | |  | |
| 78 | If the baby did not cry, breathe or move, was it born dead?  اگر بچے نے نہ حرکت کی، سانس لیا اور نہ ہی رویا تو کیا بچہ مردہ پیدا ہوا؟ | 1=Yes ہاں  2=Noنہیں  3=Don’t know پتہ نہیں  4=Not applicable | | | 🡪80  🡪80  🡪80 | |
| 79 | Was the baby macerated, that is, showed signs of decay?  کیا بچہ گلا سڑا پیدا ہوا؟ختم ہونے کے آثار۔ | 1=Yes ہاں  2=Noنہیں  3=Don’t know پتہ نہیں | | | 🡪164  🡪164  🡪164 | |
| **8. History of injuries and accident** حٓادثوں اور زخموں کی معلومات | | | | | | |
| 80 | Did the baby suffer from any injury or accident that led them to her/his death? کیا بچے کو کسی زخم یا حادثے کا سامنا کرنا پڑا جس کی وجہ سے اس کی موت ہوئی؟ | 1=Yes ہاں  2=Noنہیں  3=Don’t know پتہ نہیں | | | 🡪83  🡪83 | |
| 81 | What kind of injury or accident did the baby suffer?  بچے کو کس قسم کے زخم یا حادثے کا سامنا کرنا پڑا؟ | 1=Road traffic accident  سڑک پر ٹریفک کا حادثہ  2=Fall گر جانا  3=Drowning ڈوب جانا  4=Poisoning زہر  5=Burns جلنا  6=Violence/Assault تشد د ، زیادتی  7=Other (Specify)__________________  دیگر ( واضح کریں)  8=Don’t Know پتہ نہیں | | |  | |
| 82 | Was the injury or accident intentionally inflicted by someone else?  کیا کسی نے جان بوجھ کر زخم یا حادثہ کیا؟ | 1=Yes ہاں  2=Noنہیں  3=Don’t know پتہ نہیں | | |  | |
| 83 | Did the baby suffer from any animal/insect bite that led to her/his death?  کیا کسی جانور یا کیڑے کے کاٹنے کی وجہ سے بچے کی موت ہوئی؟ | 1=Yes ہاں  2=Noنہیں  3=Don’t know پتہ نہیں | | | 🡪85  🡪85 | |
| 84 | What type of animal or insect?  کس قسم کا جانور یا کیڑا تھا؟ | 1=Dog کتا  2=Snake سانپ  3=Insect حشرات الارض  4=Other (Specify)_____________________  ( واضح کریں)  5=Don’t know پتہ نہیں | | |  | |
| **9. Neonatal Illness History**  نومولود کی بیماری کی معلومات | | | | | | |
| 85 | Was the baby ever able to suckle or bottle-feed?  کیا بچہ دودھ پینے یا بوتل سے دودھ پینے کے قابل تھا؟ | | 1=Yes ہاں  2=Noنہیں  3=Don’t know پتہ نہیں | | 🡪90  🡪90 | |
| 86 | How soon after birth did the baby suckle or bottle-feed?  پیدائش کے کتنی دیر بعد بچے نےے دودھ پیا یا بوتل سے دودھ پیا؟ | | 1=Hours گھنٹے \|___\|___\|  2=Days دن \|___\|___\|  3=Don’t know پتہ نہیں | |  | |
| 87 | Did the baby stop suckling or bottle-feeding?  کیا بچے نے دودھ پینا یا بوتل سے دودھ پینا چھوڑ دیا؟ | | 1=Yes ہاں  2=Noنہیں  3=Don’t know پتہ نہیں | | 🡪89  🡪89 | |
| 88 | How many days after birth did the baby stop suckling or bottle-feeding?  ***00=less than 24hrs after birth***  پیدائش کے کتنے دن بعد بچے نے دودھ پینا یا بوتل سے دودھ پینا چھوڑ دیا؟ ۰۰ لکھیں اگر پیدائیش کے بعد ۲۴ گھنٹوں سے کم ہو تو۔ | | 1=Days دن \|___\|___\|  2=Don’t know پتہ نہیں | |  | |
| 89 | Was the breast feeding exclusive?  کیا بچہ صرف ماں کا دودھ پیتا تھا؟ | | 1=Yes ہاں  2=Noنہیں  3=Don’t know پتہ نہیں | |  | |
| 90 | Did the baby have convulsion?  کیا بچے کو دورے پڑے؟ | | 1=Yes ہاں  2=Noنہیں  3=Don’t know پتہ نہیں | | 🡪93  🡪93 | |
| 91 | How soon after birth did the convulsions start?  ***00=less than 24hrs after birth***  پیدائش کے کتنی دیر بعد بچے کو دورے پڑے؟  ۰۰ لکھیں اگر پیدائیش کے بعد ۲۴ گھنٹوں سے کم ہو تو۔ | | 1=Days \|___\|___\|  2=Don’t know پتہ نہیں | |  | |
| 92 | For how long did the convulsions last?  دورے کی کیفیت کب تک رہی؟ | | 1= Less than 10 minutes دس منٹ سے کم  2= Ten minutes or more دس منٹ یا زیادہ  3= Don’t know معلوم نہیں | |  | |
| 93 | Did the baby become stiff and arched backwards?  کیا بچہ اکڑا اور پیچھے کی طرف مڑ گیا تھا؟ | | 1=Yes ہاں  2=Noنہیں  3=Don’t know پتہ نہیں | |  | |
| 94 | Did s/he have stiffness of the whole body or was unable to open the mouth?  کیا اس کا پورا جسم اکڑ گیا تھا یا وہ صرف منہ کھولنے کے قابل نہیں تھا؟ | | 1=Yes ہاں  2=No نہیں  3=Don’t know معلوم نہیں | |  | |
| 95 | Did s/he have a stiff neck?  کیا اس کی گردن اکڑ گئی تھی؟ | | 1=Yes ہاں  2=No نہیں  3=Don’t know معلوم نہیں | | 🡪97  🡪97 | |
| 96 | For how long did s/he have a stiff neck?  ***00=less than 24hrs after birth***  ***اس کی گردن کتنے وقت تک اکڑی رہی۔*** ۰۰ لکھیں اگر پیدائیش کے بعد ۲۴ گھنٹوں سے کم ہو تو۔ | | 1=Days دن ۔۔۔۔۔۔۔۔۔۔۔۔۔۔۔۔۔۔۔۔۔۔۔۔۔۔۔۔۔۔۔\|___\|___\|  2=Don’t know معلوم نہیں | |  | |
| 97 | Did the child have bulging of the fontanel?  کیا بچے کا نافوخ ابھرا ہوا تھا؟ | | 1=Yes ہاں  2=Noنہیں  3=Don’t know پتہ نہیں | | 🡪99  🡪99 | |
| 98 | How many days after birth did the baby have the bulging?  ***00=less than 24hrs after birth***  پیدائش کے کتنے دن بعد بچے کا نافوخ ابھرا؟ ۰۰ لکھیں اگر پیدائیش کے بعد ۲۴ گھنٹوں سے کم ہو تو۔ | | 1=Days دن \|___\|___\|  2=Don’t know پتہ نہیں | |  | |
| 99 | Was the fontanel of the baby sunken?  کیا بچے کا نافوخ دھنسا ہوا تھا؟ | | 1=Yes ہاں  2=No نہیں  3=Don’t know معلوم نہیں | |  | |
| 100 | Did the baby become unresponsive or unconscious?  کیا بچہ بے حس یا نیم بے ہوش تھا؟ | | 1=Yes ہاں  2=Noنہیں  3=Don’t know پتہ نہیں | | 🡪104  🡪104 | |
| 101 | Was s/he unconscious for more than 24 hours?  کیا وہ چوبیس گھنٹوں سے زیادہ نیم بیہوش رہا؟ | | 1=Yes ہاں  2=No نہیں  3=Don’t know معلوم نہیں | |  | |
| 102 | Did the unconsciousness start suddenly, quickly within a single day, or slowly over many days?  کیا نیم بیہوشی اچانک شروع ہوئی۔ ایک دن میں تیزی سے ہوئی یا آہستہ آہستہ کافی دنوں میں ہوئی؟ | | 1=Suddenly اچانک  2=Yes quickly within a single day  ہاں ایک دن میں تیزی سے ہوئی۔  3=Slowly over many days  آہستہ آہستہ کافی دنوں میں ہوئی۔  4=Don’t know معلوم نہیں | |  | |
| 103 | How many days after birth did the baby become unresponsive and unconscious?  ***00=less than 24hrs after birth***  پیدائش کے کتنے دن بعد بچہ بے حس یا نیم بے ہوش ہوا؟ ۰۰ لکھیں اگر پیدائیش کے بعد ۲۴ گھنٹوں سے کم ہو تو۔ | | 1=Days دن ۔۔۔۔۔۔۔۔۔۔۔۔۔۔۔۔۔۔۔۔۔۔۔۔۔۔۔۔۔۔۔۔۔\|___\|___\|  2=Don’t knowمعلوم نہیں | |  | |
| 104 | Did s/he become weak and lethargic after a period of normal activity?  کیا وہ نارمل رہنے کے بعد کمزور یا نڈھال ہوا؟ | | 1=Yes ہاں  2=No نہیں  3=Don’t know معلوم نہیں | |  | |
| 105 | Did s/he have any urine problems?  کیا بچے کو پیشاب کا کوئی مسئلہ تھا؟ | | 1=Yes ہاں  2=No نہیں  3=Don’t know معلوم نہیں | |  | |
| 106 | Did s/he pass no urine at all?  کیا بچے کو پیشاب بالکل نہیں آیا؟ | | 1=Yes ہاں  2=No نہیں  3=Don’t know معلوم نہیں | |  | |
| 107 | During the final illness did s/he ever pass blood in the urine?  کیاآخری بیماری میں بچے کو پیشاب میں خون آیا؟ | | 1=Yes ہاں  2=No نہیں  3=Don’t know معلوم نہیں | |  | |
| 108 | Did s/he have mouth sores or white patches in the mouth or on the tongue?  کیا اس کے منہ میں چھالے، منہ یا زبان پہ سفید دھبے تھے؟ | | 1=Yes ہاں  2=No نہیں  3=Don’t know معلوم نہیں | |  | |
| 109 | Did s/he have sunken eyes?  کیا اس کی آنکھیں اندر کو دھنسی ہوئی تھیں؟ | | 1=Yes ہاں  2=No نہیں  3=Don’t know معلوم نہیں | |  | |
| 110 | During the illness that led to death did the baby bleed from anywhere?  اس بیماری کے دوران جو موت کی وجہ بنی کیا بچے کا کہیں سے خون خارج ہوا تھا؟ | | 1=Yes ہاں  2=No نہیں  3=Don’t know معلوم نہیں | | 🡪113  🡪113 | |
| 111 | Did s/he bleed from the nose, mouth or anus?  کیا اسکے ناک منہ یا مقعد سے خون آیا؟ | | 1=Yes ہاں  2=No نہیں  3=Don’t know معلوم نہیں | |  | |
| 112 | If the bleeding was from anywhere else, please record  اگر کہیں اور سے خون آیا ۔۔۔۔۔برائے مہربانی درج کریں۔ | | ____________________________________  ____________________________________ | |  | |
| 113 | Did the baby have fever?  کیا بچے کو بخار ہوا؟ | | 1=Yes ہاں  2=Noنہیں  3=Don’t know پتہ نہیں | | 🡪116  🡪116 | |
| 114 | How many days after birth did the baby have a fever?  ***00=less than 24hrs after birth***  پیدائش کے کتنے دن بعد بچے کو بخار ہوا؟  ۰۰ لکھیں اگر پیدائیش کے بعد ۲۴ گھنٹوں سے کم ہو تو۔ | | 1=Daysدن \|___\|___\|  2=Don’t know پتہ نہیں 88 | |  | |
| 115 | How many days did the fever last (OR for how long did s/he have a fever; **in days**)? ***00=less than 24hrs***  بخار کتنے دن تک رہا یا بچے کو بخار کتنے دن تک ہوا۔ (دنوں میں لکھیں) ۰۰ لکھیں اگر پیدائیش کے بعد ۲۴ گھنٹوں سے کم ہو تو۔ | | 1=Days ۔۔۔۔۔۔۔۔۔۔ دن ۔۔۔۔۔۔۔۔۔۔۔۔۔۔۔۔۔۔\|___\|___\|  2=Don’t know معلوم نہیں  3=Not applicable قابل اطلاق نہیں | |  | |
| 116 | Did the baby become cold to the touch?  کیا بچہ چھونے پر ٹھنڈا تھا؟ | | 1=Yes ہاں  2=Noنہیں  3=Don’t know پتہ نہیں | | 🡪118  🡪118 | |
| 117 | How many days after birth did the baby become cold to touch?  ***00=less than 24hrs after birth***  پیدائش کے کتنے دن بعد بچہ چھونے پر ٹھنڈا ہوا؟ ۰۰ لکھیں اگر پیدائیش کے بعد ۲۴ گھنٹوں سے کم ہو تو۔ | | 1=Days دن \|___\|___\|  2=Don’t know پتہ نہیں | |  | |
| 118 | Did the baby have a cough?  کیا بچے کو کھانسی ہوئی؟ | | 1=Yes ہاں  2=Noنہیں  3=Don’t know پتہ نہیں | | 🡪123  🡪123 | |
| 119 | How many days after birth did the baby start to cough?  ***00=less than 24hrs***  پیدائش کے کتنے دن بعد بچے کو کھانسی ہوئی؟ ۰۰ لکھیں اگر پیدائیش کے بعد ۲۴ گھنٹوں سے کم ہو تو | | 1=Days دن \|___\|___\|  2=Don’t know پتہ نہیں | |  | |
| 120 | How long before death did this cough start?  وفات سے کتنی دیر پہلے کھانسی شروع ہوئی؟ | | 1=On the same day of death  وفات والے دن  2=1-3 days before death  وفات کے ۱ سے ۳ دن پہلے  3=4-7 days before death  وفات کے ۴ سے ۷ دن پہلے  4=More than 1 week but within the month  ۱ ہفتے سے زیادہ مگر ایک مہینے سے کم۔  5=Don’t know معلوم نہیں | |  | |
| 121 | For how long did s/he have a cough (in days)?  ***00=less than 24hrs after birth***  اسکو کھانسی کتنا وقت رہی۔ ۰۰ لکھیں اگر پیدائیش کے بعد ۲۴ گھنٹوں سے کم ہو تو۔ | | 1=Days ۔۔۔۔۔۔۔۔۔۔۔ دن ۔۔۔۔۔۔۔۔۔\|___\|___\|  2=Don’t knowمعلوم نہیں | |  | |
| 122 | Did s/he make a whooping sound when coughing?  کیا کھانسی کرتے وقت اس کے حلق سے آواز آتی تھی؟ | | 1=Yes ہاں  2=No نہیں  3=Don’t know معلوم نہیں | |  | |
| 123 | Did the baby have fast breathing?  کیا بچے کی سانس تیز تھی؟ | | 1=Yes ہاں  2=Noنہیں  3=Don’t know پتہ نہیں | | 🡪126  🡪126 | |
| 124 | How many days after birth did the baby start breathing fast?  ***00=less than 24hrs after birth***  پیدائش کے کتنے دن بعد بچے کی سانس تیز ہوئی؟ ۰۰ لکھیں اگر پیدائیش کے بعد ۲۴ گھنٹوں سے کم ہو تو۔ | | 1=Days دن \|___\|___\|  2=Don’t know پتہ نہیں | |  | |
| 125 | For how long did s/he have the fast breathing (in days)?  ***00=less than 24hrs after birth***  کتنے وقت تک اس کی سانس تیز رہی (دنوں میں)۔  ۰۰ لکھیں اگر پیدائیش کے بعد ۲۴ گھنٹوں سے کم ہو تو۔ | | 1=Days ۔۔۔۔۔۔۔۔۔۔۔ دن ۔۔۔۔۔۔۔۔۔\|___\|___\|  2=Don’t knowمعلوم نہیں | |  | |
| 126 | Did the baby have difficulty breathing?  کیا بچے کو سانس لینے میں دشواری تھی؟ | | 1=Yes ہاں  2=Noنہیں  3=Don’t know پتہ نہیں | | 🡪129  🡪129 | |
| 127 | How many days after birth did the baby start having difficulty in breathing?  ***00=less than 24hrs after birth***  پیدائش کے کتنے دن بعد بچے کو سانس لینے میں دشواری ہوئی؟ ۰۰ لکھیں اگر پیدائیش کے بعد ۲۴ گھنٹوں سے کم ہو تو۔ | | 1=Daysدن \|___\|___\|  2=Don’t know پتہ نہیں | |  | |
| 128 | For how long did s/he have the breathing problem (in days)?  ***00=less than 24hrs after birth***  کتنے وقت تک اسکو سانس لینے میں دشواری ہوئی۔ ۰۰ لکھیں اگر پیدائیش کے بعد ۲۴ گھنٹوں سے کم ہو تو | | 1=Days ۔۔۔۔۔۔۔۔۔۔۔ دن ۔۔۔۔۔۔۔۔۔\|___\|___\|  2=Don’t knowمعلوم نہیں | |  | |
| 129 | Did the baby have chest -indrawing?  کیا بچے کی پسلیاں اندر کی طرف چلیں؟ | | 1=Yes ہاں  2=Noنہیں  3=Don’t know پتہ نہیں | | 🡪131  🡪131 | |
| 130 | For how long did s/he have the chest in drawing (in days)?  ***00=less than 24hrs after birth***  کتنے وقت تک اس کی پسلیاں اندر کی طرف چلیں۔ (دنوں میں لکھیں) ۰۰ لکھیں اگر پیدائیش کے بعد ۲۴ گھنٹوں سے کم ہو تو | | 1=Days ۔۔۔۔۔۔۔۔۔۔۔ دن ۔۔۔۔۔۔۔۔۔\|___\|___\|  2=Don’t knowمعلوم نہیں | |  | |
| 131 | Did the baby have grunting?  DEMONSTRATE  کیا بچہ غرغرایا؟  ظاہر کر کے دکھائیں۔ | | 1=Yes ہاں  2=Noنہیں  3=Don’t know پتہ نہیں | |  | |
| 132 | Did the baby have flaring of the nostrils?  کیا بچے کے نتھنے پھڑپھڑائے؟ | | 1=Yes ہاں  2=Noنہیں  3=Don’t know پتہ نہیں | |  | |
| 133 | Did s/he have yellow discoloration of the eyes?  **کیا اس کی آنکھوں کی رنگت زردی مائل تھی؟** | | 1=Yes ہاں  2=No نہیں  3=Don’t know معلوم نہیں | | 🡪136  🡪136 | |
| 134 | For how long did s/he have the yellow discoloration of the eyes?  ***00=less than 24hrs after birth***  ***کتنے وقت تک اس کی آنکھیں زردی مائل رہیں؟*** ۰۰ لکھیں اگر پیدائیش کے بعد ۲۴ گھنٹوں سے کم ہو تو | | 1=Days ۔۔۔۔۔۔۔۔۔۔۔ دن ۔۔۔۔۔۔۔۔۔\|___\|___\|  2=Don’t knowمعلوم نہیں | |  | |
| 135 | When did this yellowish discoloration start? ***00=less than 24hrs after birth***  ***زردی مائل رنگت کب شروع ہوئی؟*** ۰۰ لکھیں اگر پیدائیش کے بعد ۲۴ گھنٹوں سے کم ہو تو | | 1=Days ۔۔۔۔۔۔۔۔۔۔۔ دن ۔۔۔۔۔۔۔۔۔\|___\|___\|  2=Don’t knowمعلوم نہیں | |  | |
| 136 | Did the baby have diarrhea?  کیا بچے کو اسہال ہوا؟ | | 1=Yes ہاں  2=Noنہیں  3=Don’t know پتہ نہیں | | 🡪142  🡪142 | |
| 137 | For how long did s/he have the diarrhea (in days)? ***00=less than 24hrs after birth***  ***کتنے وقت تک اسکو اسہال رہا؟*** ۰۰ لکھیں اگر پیدائیش کے بعد ۲۴ گھنٹوں سے کم ہو تو | | 1=Days ۔۔۔۔۔۔۔۔۔۔۔ دن ۔۔۔۔۔۔۔۔۔\|___\|___\|  2=Don’t knowمعلوم نہیں | |  | |
| 138 | How many days after birth did the baby have diarrhoea?  پیدائش کے کتنے دن بعد بچے کو اسہال ہوا؟ | | 1=Days دن \|___\|___\|  2=Don’t know پتہ نہیں | |  | |
| 139 | How long before death did this diarrhea start? ***00=less than 24hrs after birth***  ***وفات سے کتنی دیر پہلے اسہال ہوا؟*** ۰۰ لکھیں اگر پیدائیش کے بعد ۲۴ گھنٹوں سے کم ہو تو | | 1=Days ۔۔۔۔۔۔۔۔۔۔۔ دن ۔۔۔۔۔۔۔۔۔\|___\|___\|  2=Don’t knowمعلوم نہیں | |  | |
| 140 | When the diarrhoea was most severe, how many times did the baby pass stools in a day?  جب اسہال شدید تھا، تو بچہ دن میں کتنی مرتبہ دست کرتا تھا؟ | | 1=Number تعداد \|___\|___\|  2=Don’t know پتہ نہیں | |  | |
| 141 | Was there blood in the stool?  کیا دست میں خون تھا؟ | | 1=Yes ہاں  2=Noنہیں  3=Don’t know پتہ نہیں | |  | |
| 142 | Did the baby have vomiting?  کیا بچے کو الٹیاں تھیں؟ | | 1=Yes ہاں  2=Noنہیں  3=Don’t know پتہ نہیں | | 🡪146  🡪146 | |
| 143 | How many days after birth did vomiting start?  ***00=less than 24hrs after birth***  پیدائش کے کتنے دن بعد بچے کو الٹیاں ہوئیں؟ ۰۰ لکھیں اگر پیدائیش کے بعد ۲۴ گھنٹوں سے کم ہو تو۔ | | 1=Days دن \|___\|___\|  2=Don’t know پتہ نہیں | |  | |
| 144 | When the vomiting was most severe, how many times did the baby vomit in a day?  جب الٹیاں شدید تھیں، تو بچہ دن میں کتنی مرتبہ  الٹی کرتا تھا؟ | | 1=Number of times a day \|___\|___\|  ایک دن میں کتنی مرتبہ  2=Don’t know پتہ نہیں | |  | |
| 145 | Did s/he vomit "coffee grounds" or bright red/blood?  کیا الٹی کا رنگ سرخی مائل بھورا یا تیز سرخ تھا؟ | | 1=Yes ہاں  2=No نہیں  3=Don’t know معلوم نہیں | |  | |
| 146 | Did the baby have abdominal distension?  کیا بچے کا پیٹ پھولا ہوا تھا؟ | | 1=Yes ہاں  2=Noنہیں  3=Don’t know پتہ نہیں | | 🡪149  🡪149 | |
| 147 | How many days after birth did the baby have abdominal distension?  ***00=less than 24hrs after birth***  پیدائش کے کتنے دن بعد بچے کا پیٹ پھولا؟ ۰۰ لکھیں اگر پیدائیش کے بعد ۲۴ گھنٹوں سے کم ہو تو | | 1=Days دن \|___\|___\|  2=Don’t know پتہ نہیں | |  | |
| 148 | For how long did s/he have more than usual protruding belly? ***00=less than 24hrs after birth***  ***کتنی دیر تک اس کا پیٹ معمول کے ابھرے ہوئے پیٹ سے زیادہ پھولا رہا؟*** ۰۰ لکھیں اگر پیدائیش کے بعد ۲۴ گھنٹوں سے کم ہو تو | | 1=Days ۔۔۔۔۔۔۔۔۔۔۔ دن ۔۔۔۔۔۔۔۔۔\|___\|___\|  2=Don’t knowمعلوم نہیں | |  | |
| 149 | Did s/he have any mass in the abdomen?  کیا اس کے پیٹ میں گولا سا بنا ہوا تھا؟ | | 1=Yes ہاں  2=No نہیں  3=Don’t know معلوم نہیں | | 🡪151  🡪151 | |
| 150 | For how long did s/he have the mass in the abdomen?  ***00=less than 24hrs after birth***  کتنے وقت تک اس کے پیٹ میں گولا بنا رہا؟ ۰۰ لکھیں اگر پیدائیش کے بعد ۲۴ گھنٹوں سے کم ہو تو | | 1=Days ۔۔۔۔۔۔۔۔۔۔۔ دن ۔۔۔۔۔۔۔۔۔\|___\|___\|  2=Don’t knowمعلوم نہیں | |  | |
| 151 | During the illness that led to death, did the baby have pus discharging from the umbilical cord stump?  کیا اس بیماری کے دوران جو موت کی وجہ بنی، بچے کے ناڑو سے پیپ کا اخراج ہوا تھا؟ | | 1=Yes ہاں  2=No نہیں  3=Don’t know معلوم نہیں | |  | |
| 152 | During the illness that led to death, did the baby have redness of the umbilical cord stump?  کیا اس بیماری کے دوران جو موت کی وجہ بنی، بچے کا ناڑو سرخ ہوا تھا؟ | | 1=Yes ہاں  2=No نہیں  3=Don’t know معلوم نہیں | |  | |
| 153 | Did s/he have any skin problems?  کیا اسے جلد کا کوئی مسئلہ ہوا تھا؟ | | 1=Yes ہاں  2=No نہیں  3=Don’t know معلوم نہیں | | 🡪161  🡪161 | |
| 154 | Did s/he have any ulcers, abscess or sores anywhere except the feet during the illness that led to death?  کیا اس بیماری کے دوران جو موت کی وجہ بنی، اس کے پاوں کے علاوہ کہیں بھی السر، پھوڑے یا چھالے تھے؟ | | 1=Yes ہاں  2=No نہیں  3=Don’t know معلوم نہیں | |  | |
| 155 | Did s/he have any ulcers, abscess or sores on the feet during the illness that led to death?  کیا اس بیماری کے دوران جو موت کی وجہ بنی، اس کے پاوں پر السر، پھوڑے یا چھالے تھے؟ | | 1=Yes ہاں  2=No نہیں  3=Don’t know معلوم نہیں | |  | |
| 156 | During the illness that led to death, did s/he have any skin rash?  کیا اس بیماری کے دوران جو موت کی وجہ بنی، اسکی جلد پر خارش کا مسئلہ ہوا؟ | | 1=Yes ہاں  2=No نہیں  3=Don’t know معلوم نہیں | |  | |
| 157 | During the illness that led to death, did the baby have skin bumps containing pus or a single area with pus?  کیا اس بیماری کے دوران جو موت کی وجہ بنی، اسکی جلد پر یا کسی بھی ایک حصے پہ پیپ سے بھرے دانے تھے؟ | | 1=Yes ہاں  2=No نہیں  3=Don’t know معلوم نہیں | | 🡪159  🡪159 | |
| 158 | For how long did s/he have the skin bumps containing pus or a single area with pus?  ***00=less than 24hrs after birth***  ***کتنے وقت تک اسکی جلد پر یا کسی بھی ایک حصے پر پیپ سے بھرے دانے ہوئے تھے؟*** ۰۰ لکھیں اگر پیدائیش کے بعد ۲۴ گھنٹوں سے کم ہو تو۔ | | 1=Days ۔۔۔۔۔۔۔۔۔۔۔ دن ۔۔۔۔۔۔۔۔۔\|___\|___\|  2=Don’t knowمعلوم نہیں | |  | |
| 159 | During the illness that led to death, did the baby have areas of skin with redness and swelling?  کیا اس بیماری کے دوران جو موت کی وجہ بنی، بچے کی جلد پر سرخی یا سوزش تھی؟ | | 1=Yes ہاں  2=No نہیں  3=Don’t know معلوم نہیں | |  | |
| 160 | During the illness that led to death, did the baby have areas of skin that turned black?  کیا اس بیماری کے دوران جو موت کی وجہ بنی، بچے کی جلد کا کوئی حصہ کالا پڑ گیا تھا؟ | | 1=Yes ہاں  2=No نہیں  3=Don’t know معلوم نہیں | |  | |
| 161 | Did the baby have yellow palms and soles?  کیا بچے کی ہتھیلیاں اور تلوے زرد تھے؟ | | 1=Yes ہاں  2=Noنہیں  3=Don’t know پتہ نہیں | | 🡪164  🡪164 | |
| 162 | How many days after birth did the yellow palms and soles begin?  ***00=less than 24hrs after birth***  پیدائش کے کتنے دن بعد بچے کی ہتھیلیاں اور تلوے زردہوئے؟ ۰۰ لکھیں اگر پیدائیش کے بعد ۲۴ گھنٹوں سے کم ہو تو۔ | | 1=Days دن \|___\|___\|  2=Don’t know پتہ نہیں | |  | |
| 163 | For how many days did the baby have yellow palms or soles?  ***00=less than 24hrs after birth***  کتنے دن تک بچے کی ہتھیلیاں اور تلوے زرد رہے؟ ۰۰ لکھیں اگر پیدائیش کے بعد ۲۴ گھنٹوں سے کم ہو تو۔ | | 1=Days دن \|___\|___\|  2=Don’t know پتہ نہیں | |  | |
| **10. Mother’s health and contextual factors** ماں کی صحت اور سياق عبارت سے متعلق اجزا | | | | | | |
| 164 | What was the age of the mother at the time the baby died?  بچے کی وفات کے وقت ماں کی کیا عمر تھی؟ | 1=Years سال \|___\|___\|  2=Don’t know پتہ نہیں | | |  | |
| 165 | Did the mother receive antenatal care?  کیا ماں نے حمل کے دوران معائنہ کروایا؟ | 1=Yes ہاں  2=Noنہیں  3=Don’t know پتہ نہیں | | |  | |
| 166 | Did the mother receive tetanus toxoid (TT) vaccine?  کیا ماں کو تشنج سے بچاؤ کی ویکسین لگی؟ | 1=Yes ہاں  2=Noنہیں  3=Don’t know پتہ نہیں | | | 🡪168  🡪168 | |
| 167 | How many doses?  کتنی مرتبہ؟ | 1=Number of doses تعداد \|___\|___\|  2=Don’t know پتہ نہیں | | |  | |
| 168 | Was the mother ever tested for HIV?  کیا ماں نے کبھی ایچ آئی وی ٹیسٹ کروایا تھا؟ | 1=Yes ہاں  2=No نہیں  3=Don’t know معلوم نہیں | | | 🡪171  🡪171 | |
| 169 | Was the HIV test for the mother ever positive?  کیا ماں کا ایچ آئی وی ٹیسٹ کبھی مثبت آیا؟ | 1=Yes ہاں  2=No نہیں  3=Don’t know معلوم نہیں | | |  | |
| 170 | Has the deceased’s (biological) mother ever been told she has “AIDS” by a health worker?  کیا ہیلتھ ورکر نے کبھی متاثرہ ماں کو بتایا تھا کہ اسے ایڈز ہے؟ | 1=Yes ہاں  2=No نہیں  3=Don’t know معلوم نہیں | | |  | |
| 171 | How is the mother’s health now  ماں کی صحت اب کیسی ہے؟ | 1=Healthy صحتمند  2=Sick بیمار  3=Not alive مردہ  4=Don’t know پتہ نہیں | | | 🡪174  🡪174 | |
| 172 | Did the mother die during or after the delivery?  کیا ماں زچگی کے دوران یا اس کے بعد وفات پا گئی؟ | 1=During delivery زچگی کے دوران  2=After deliveryزچگی کے بعد  3=Don’t knowمعلوم نہیں | | | 🡪174  🡪174 | |
| 173 | How long after birth did the mother die?  ***00=less than 24hrs after birth***  پیدائیش کے کتنے وقت بعد ماں نے وفات پائی؟ ۰۰ لکھیں اگر پیدائیش کے بعد ۲۴ گھنٹوں سے کم ہو تو۔ | 1=Days ۔۔۔۔۔۔۔۔۔۔۔ دن ۔۔۔۔۔۔۔۔۔\|___\|___\|  2=Don’t knowمعلوم نہیں | | |  | |
| **11. Treatment and health service use for the final illness** آخری بیماری کا علاج اور صحت کی سہولیات | | | | | | |
| 174 | Did the baby receive any treatment for the illness that led to death  جس بیماری کی وجہ سے بچے کی موت ہوئی کیا اس کیلئے علاج کروایا؟ | 1=Yes ہاں  2=Noنہیں  3=Don’t know پتہ نہیں | | | 🡪184  🡪184 | |
| 175 | Can you please list the treatments the baby was given for the illness that led to death?  کیا آپ وجہ الموت بیماری کے علاج کی فہرست بتا سکتی ہیں؟  COPY FROM PRESCRIPTION/DISCHARGE NOTES IF AVAILABLE  اگرنسخے، یا ہسپتال کے اخراج کی پرچی موجود ہے تو اس سے نقل کریں | _________________________________  _________________________________  _________________________________ | | |  | |
| 176 | Please tell me at which of the following places or facilities the baby received treatment during the illness that led to death  برائے مہربانی ان سب جگہوں اور سہولیات کے بارے میں بتائیں جو وجہ الموت بیماری کے علاج کےلئے استعمال ہوئیں؟ | 1= Home گھر  2= Traditional healer حکیم  3= Government clinic سرکاری کلینک  4= Government Hospital سرکاری ہسپتال  5= Private clinic پرائیویٹ کلینک  6= Private Hospital پرائیویٹ ہسپتال  7= Pharmacy, Drug seller, Store  فارمیسی، دوائی بیچنے والا، سٹور  8= Other  (Specify)_____________________  دیگر ( واضح کریں)  9= Don't know پتہ نہیں | | |  | |
| 177 | Did s/he receive treatment with oral rehydration salts?  کیا اس کا علاج او آر ایس سے کیا گیا؟ | 1=Yes ہاں  2=No نہیں  3=Don’t know معلوم نہیں | | |  | |
| 178 | Did s/he receive intravenous fluids (drip) treatment?  کیا اس کا علاج ڈرپ لگا کر کیا گیا؟ | 1=Yes ہاں  2=No نہیں  3=Don’t know معلوم نہیں | | |  | |
| 179 | Did s/he receive a blood transfusion?  کیا اسے خون لگا؟ | 1=Yes ہاں  2=No نہیں  3=Don’t know معلوم نہیں | | |  | |
| 180 | Did s/he receive treatment or food through a tube passed through the nose?  کیا اسے علاج یا خوراک ناک کی نالی کے زریعے دی گئی؟ | 1=Yes ہاں  2=No نہیں  3=Don’t know معلوم نہیں | | |  | |
| 181 | Did s/he need injectable (IM or IV) antibiotics?  کیا اسے جراثیم کش ادویات کی ضرورت تھی؟ | 1=Yes ہاں  2=No نہیں  3=Don’t know معلوم نہیں | | |  | |
| 182 | Did s/he receive any other treatment?  کیا اسکا کوئی اور علاج ہوا؟ | 1=Yes ہاں  2=No نہیں  3=Don’t know معلوم نہیں | | |  | |
| 183 | If yes to other treatment, please specify.  اگر کوئی اور علاج ہوا ہے تو برائے مہربانی وضاحت کریں۔ | _____________________________________  _____________________________________  _____________________________________ | | |  | |
| 184 | Did s/he have an operation for the illness?  کیا بیماری کی وجہ سے اسکا آپریشن ہوا تھا؟ | 1=Yes ہاں  2=No نہیں  3=Don’t know معلوم نہیں | | |  | |
| 185 | Did s/he have any operation before death?  کیا مرنے سے پہلے اسکا کوئی آپریشن ہوا تھا؟ | 1=Yes ہاں  2=No نہیں  3=Don’t know معلوم نہیں | | |  | |
| 186 | Was s/he discharged from hospital very ill?  کیا اسے بہت علیل حالت میں ہسپتال سے فارغ کیا گیا تھا؟ | 1=Yes ہاں  2=No نہیں  3=Don’t know معلوم نہیں | | |  | |
| 187 | In the final days did anyone travel with him/her to hospital or health facility?  کیا آخری دنوں میں اس کے ساتھ کسی نے ہسپتال یا مرکز صحت کی طرف سفر کیا تھا؟ | 1=Yes ہاں  2=No نہیں  3=Don’t know معلوم نہیں | | |  | |
| 188 | Was motorized transport used to get him/her to the hospital or health facility?  کیا اسے ہسپتال یا مرکز صحت لیجانے کیلیئے موٹر کار کا استعمال کیا گیا؟ | 1=Yes ہاں  2=No نہیں  3=Don’t know معلوم نہیں | | |  | |
| 189 | Were there any problems during admission to the hospital or health facility?  کیا ہسپتال یا مرکز صحت میں داخلے کے وقت کوئی مسائل پیش آئے؟ | 1=Yes ہاں  2=No نہیں  3=Don’t know معلوم نہیں | | |  | |
| 190 | Were there any problems with the way s/he was treated at the hospital or health facility?  کیا ہسپتال یا مرکز صحت میں علاج کے حوالے سے اسے کوئی مسائل پیش آئے؟ | 1=Yes ہاں  2=No نہیں  3=Don’t know معلوم نہیں | | |  | |
| 191 | Were there any problems getting medications or diagnostic tests in the hospital or health facility?  کیا ہسپتال یا مرکز صحت میں تشخیصی ٹیسٹ یا ادویات حاصل کرنے میں مسائل پیش آئے؟ | 1=Yes ہاں  2=No نہیں  3=Don’t know معلوم نہیں | | |  | |
| 192 | Does it take more than 2 hours to get to the nearest hospital or health facility from [NAME’s] household?  کیا گھر سے نزدیکی ہسپتال یا مرکز صحت لیجاتے ہوئے اسے دو گھنٹے سے زیادہ وقت لگا؟ | 1=Yes ہاں  2=No نہیں  3=Don’t know معلوم نہیں | | |  | |
| 193 | Was traditional medicine used?  کیا روائیتی ادویات استعمال کی گئیں؟ | 1=Yes ہاں  2=No نہیں  3=Don’t know معلوم نہیں | | |  | |
| 194 | Do you have any health records that belonged to the deceased?  کیا مرحوم (بچے) کے حوالے سے آپ کے پاس صحت کا کوئی ریکارڈ موجود ہے؟ | 1=Yes ہاں  2=No نہیں  3=Don’t know معلوم نہیں | | | 🡪196  🡪196 | |
| 195 | Can I see the health records that belonged to the deceased?  کیا میں وہ ریکارڈ دیکھ سکتی ہوں جو مرحوم بچے سے متعلق ہے؟ | 1=Yes ہاں  2=No نہیں  3=Don’t know معلوم نہیں | | |  | |
| 196 | In the month before death, how many contacts with formal health services did the baby have?  مرنے کے آخری مہینے میں،بچہ کتنی مرتبہ کارکن صحت کے پاس گیا؟ | 1=Number of contacts \|___\|___\|  کارکن صحت کی تعداد  2=Don’t know پتہ نہیں | | |  | |
| 197 | Did a health care worker tell you the cause of death?  کیا کارکن صحت نے آپ کو وجہ بتائی؟ | 1=Yes ہاں  2=Noنہیں  3=Don’t know پتہ نہیں | | |  | |
| 198 | What did the health care worker say?  کارکن صحت نے کیا کہا؟ | _________________________________  _________________________________  _________________________________ | | |  | |
| **12. Data abstracted from death certificate** موت کے تصدیق نامے سے لی گئ معلومات | | | | | | |
| 199 | Do you have a death certificate for the baby?  کیا آپ کے پاس بچے کی موت کا تصدیق نامہ ہے؟ | 1=Yes ہاں  2=Noنہیں  3=Don’t know پتہ نہیں | | | 🡪206  🡪206 | |
| 200 | Can I see the death certificate?  کیا میں موت کا تصدیق نامہ دیکھ سکتی ہوں؟  COPY DAY, MONTH AND YEAR OF DEATH FROM THE DEATH CERTIFICATE  موت کے تصدیق نامے سے موت کا دن مہینہ اور سال نقل کریں۔ | 1= \|___\|___\| \|___\|___\| \|___\|___\|  dd mm yy  2=Don't know پتہ نہیں | | |  | |
| 201 | COPY DAY, MONTH AND YEAR OF ISSUE OF DEATH CERTIFICATE  موت کے تصدیق نامے سے تصدیق نامہ جاری ہونے کا دن مہینہ اور سال نقل کریں | 1=\|___\|___\| \|___\|___\| \|___\|___\|  dd mm yy  2=Don't know پتہ نہیں | | |  | |
| 202 | RECORD THE CAUSE OF DEATH FROM THE FIRST (TOP) LINE OF THE DEATH CERTIFICATE  موت کے تصدیق نامے کی پہلی سطر سے موت کی وجہ درج کریں۔  ______________________________________________________________________________________ | | | | | |
| 203 | RECORD THE CAUSE OF DEATH FROM THE SECOND LINE OF THE DEATH CERTIFICATE (IF ANY)  موت کے تصدیق نامے کی دوسری سطر سے موت کی وجہ درج کریں( اگر کوئی ہے)۔  ______________________________________________________________________________________ | | | | | |
| 204 | RECORD THE CAUSE OF DEATH FROM THE THIRD LINE OF THE DEATH CERTIFICATE (IF ANY)  موت کے تصدیق نامے کی تیسری سطر سے موت کی وجہ درج کریں( اگر کوئی ہے)۔  ______________________________________________________________________________________ | | | | | |
| 205 | RECORD THE CAUSE OF DEATH FROM THE FOURTH LINE OF THE DEATH CERTIFICATE (IF ANY)  موت کے تصدیق نامے کی چوتھی سطر سے موت کی وجہ درج کریں( اگر کوئی ہے)۔  ______________________________________________________________________________________ | | | | | |
| **13. Data abstracted from other health records** صحت کے دوسرے ریکارڈ سے لی گئی معلومات | | | | | | |
| 206 | Other health records available  کیاصحت کے دوسرے دستاویزات موجود تھے | 1=Yes ہاں  2=No نہیں | | 🡪216 | | |
| 207 | For each type of health record summarizes details for last 2 visits (if more than 2) and record date of issue. (record information about mother and stillborn deceased child) کسی بھی صحت سے متعلقہ ریکارڈ موجود ہونے کی صورت میں آخری دو معائنوں کا خلاصہ لکھیں ( اگر دو سے زیادہ ہوں تو) اور تاریخ کا اندراج کریں۔ ( مرحوم اور ماں کے بارے میں معلومات درج کریں) | | | | | |
| 208 | Burial Permit (Cause of death)تدفین کا اجازت نامہ( موت کی وجہ) _____________________________________________________________  ______________________________________________________________________________________ | | | | | |
| 209 | Post mortem Results (Cause of death) تشريح الجسہ کا نتیجہ ( موت کی وجہ) ________________________________________________________  ______________________________________________________________________________________ | | | | | |
| 210 | MCH/ANC Card (Relevant information) ماں اور بچے کی صحت،دوران حمل کا کارڈ( متعلقہ معلومات_______________________________________________________  ______________________________________________________________________________________ | | | | | |
| 211 | Hospital Prescription (Relevant information) ہسپتال کا نسخہ( متعلقہ معلومات) __________________________________________________  ______________________________________________________________________________________ | | | | | |
| 212 | Treatment Cards (Relevant information)علاج کے کارڈ( متعلقہ معلومات) _____________________________________________________  ______________________________________________________________________________________ | | | | | |
| 213 | Hospital Discharge (Relevant information)ہسپتال سے اخراج( متعلقہ معلومات) ____________________________________________________  ______________________________________________________________________________________ | | | | | |
| 214 | Laboratory Results (Relevant information) لیبارٹری کے نتائج ( متعلقہ معلومات) ____________________________________________________  ______________________________________________________________________________________ | | | | | |
| 215 | Other Hospital Documents ہسپتال کے دوسرے دستاویزات Specify:____________________ ______________________________  ______________________________________________________________________________________واضح کریں | | | | | |
| 216 | RECORD THE TIME AT THE END OF INTERVIEW  انٹرویو کے اختتام پر وقت ریکارڈ کریں | Hours گھنٹے \|___\|___\|  Minutes منٹس \|___\|___\| | | | | |

| Interviewers observations انٹرویو لینے والے کے مشاہدات  To be filled in after completing interview  انٹرویو مکمل ہونے کے بعد لکھیں |
| --- |
| _____________________________________________________________________________________  _____________________________________________________________________________________  _____________________________________________________________________________________  _____________________________________________________________________________________  _____________________________________________________________________________________ |
| comments on specific questions خا ص سوالات کے بارے میں رائے  _____________________________________________________________________________________  _____________________________________________________________________________________  _____________________________________________________________________________________  _____________________________________________________________________________________ |
| Any other comments کوئی اور رائے  _____________________________________________________________________________________  _____________________________________________________________________________________  _____________________________________________________________________________________  _____________________________________________________________________________________  Name and ID of the Interviewer انٹرویو لینے والی کا نام اور شناخت نمبر  _____________________________________  Signature: __________________________دستخط  Date تاریخ \|___\|___\|/\|___\|____\|/\|____\|____\| |
| Supervisors Notes (following review of form) سپروائزر کے مشاہدات  _____________________________________________________________________________________  _____________________________________________________________________________________  _____________________________________________________________________________________  _____________________________________________________________________________________ |
| Name and signature of the supervisor سپر وائز کا نام اور دستخط _____________________________________  Date تاریخ \|___\|___\|/\|___\|____\|/\|____\|____\| |
